# Identification and characterization of a small-molecule metallophore involved in lanthanide metabolism

**DOI:** 10.1073/pnas.2322096121

**Published:** 2024-07-30

**Authors:** Alexa M. Zytnick, Sophie M. Gutenthaler-Tietze, Allegra T. Aron, Zachary L. Reitz, Manh Tri Phi, Nathan M. Good, Daniel Petras, Lena J. Daumann, Norma Cecilia Martinez-Gomez

**Affiliations:** ^a^Department of Plant and Microbial Biology, University of California, Berkeley, Berkeley, CA 94720; ^b^Department of Chemistry, Ludwig-Maximilians-Universität München, Munich 81377, Germany; ^c^Chair of Bioinorganic Chemistry, Heinrich-Heine-Universität Düsseldorf, Düsseldorf 40225, Germany; ^d^Department of Chemistry and Biochemistry, University of Denver, Denver, CO 80210; ^e^Bioinformatics Group, Wageningen University, Wageningen 6708PB, The Netherlands; ^f^Department of Ecology, Evolution and Marine Biology, University of California, Santa Barbara, CA 93117; ^g^Interfaculty Institute of Microbiology and Medicine, Universität Tübingen, Tübingen 72074, Germany

**Keywords:** lanthanides, bioavailability, bioaccumulation, metallophores

## Abstract

Many bacteria secrete metallophores, low-molecular-weight organic compounds that bind ions with high selectivity and affinity, in order to access essential metals from the environment. Previous work has elucidated the structures and biosynthetic machinery of metallophores specific for iron, zinc, nickel, molybdenum, and copper. No physiologically relevant lanthanide-binding metallophore has been discovered despite the knowledge that lanthanide metals (Ln) have been revealed to be essential cofactors for certain alcohol dehydrogenases across a diverse range of phyla. Here, we report the biosynthetic machinery, the structure, and the physiological relevance of a lanthanophore, methylolanthanin. The structure of methylolanthanin exhibits a unique 4-hydroxybenzoate moiety which has not previously been described in other metallophores. We find that production of methylolanthanin is required for normal levels of Ln accumulation in the methylotrophic bacterium *Methylobacterium extorquens* AM1, while overexpression of the molecule greatly increases bioaccumulation and adsorption. Our results provide a clearer understanding of how Ln-utilizing bacteria sense, scavenge, and store Ln; essential processes in the environment where Ln are poorly bioavailable. More broadly, the identification of this lanthanophore opens doors for study of how biosynthetic gene clusters are repurposed for additional functions and the complex relationship between metal homeostasis and fitness.

## Significance

The production of metallophores is essential for microorganisms to survive in metal-limiting natural environments. With the first lanthanide-dependent enzyme, XoxF, being discovered just a decade ago, lanthanides have recently joined the class of life metals. Here, we characterize methylolanthanin, a lanthanide chelator with a unique 4-hydroxy benzoate moiety. Methylolanthanin production is essential for wild-type concentrations of cellular lanthanides. As demand for supply-chain limited lanthanide metals grow, so does interest in microbial mining methods for lanthanides; here we show that overproduction of methylolanthanin increases cellular lanthanide concentrations, making *Methylobacterium extorquens* AM1 attractive as a green chassis for lanthanide mining. Further, the identification of this molecule is an essential puzzle piece in understanding how lanthanides are sensed and acquired in nature.

Metal ions are essential for life—it is estimated that 40 to 50% of all enzymes require a metal ion for proper function (1–3). Whether serving in catalytic or structural roles, metal ions are involved in biological processes that range from respiration and DNA replication to the biosynthesis of metabolic intermediates. The importance of metal ions is underscored by the extensive systems that organisms across the entire tree of life have developed to not only sense and acquire metals from the environment but also regulate concentrations within their cells.

Many bacteria secrete metallophores, small-molecule chelators, to make environmental metals more bioavailable (2). Siderophores (Fe), chalkophores (Cu), zincophores (Zn), molybdophores (Mo), and nickelophores (Ni) are metallophores that have been reported (4). Metallophore structures are categorized based on their metal-binding moieties—such as catecholates, phenolates, hydroxamates, carboxylates, and diazeniumdiolates—each of which have unique physiochemical properties (5, 6). The majority of known metallophores are biosynthesized by nonribosomal peptide synthetases (NRPSs) or NRPS-independent siderophore (NIS) synthetases. Metallophore biosynthetic gene clusters (BGCs) often include genes encoding transport systems that bring the metallophore–metal complex into the cell for metal release. In Gram-negative bacteria, TonB-complexes provide the energy for transport into the periplasm via beta-barrel outer membrane receptors. Depending on the metallophore system, the complex may remain in the periplasm or be transported to the cytoplasm via an ATP-binding cassette (ABC) transporter before the metal is released by metallophore hydrolysis and/or metal reduction. As free metal in the cell can be toxic due to the formation of reactive oxygen species or through mismetallation of enzymes, metal acquisition and uptake genes are often tightly regulated by metal-responsive transcription factors such as ferric uptake regulator (Fur) and nickel uptake regulator (Nur) family proteins or by sigma factor/anti-sigma factor signaling systems (7–9).

While many are familiar with canonical *d*-block life metals such as iron, copper, and magnesium, members of the *f-*block lanthanide series have recently solidified their place among the life metals. Lanthanides were found to be biologically relevant in 2011 when it was discovered that MxaFI, a pyrroloquinoline quinone (PQQ) and Ca-dependent methanol dehydrogenase from the methylotrophic bacterium *Methylobacterium extorquens* AM1, had a Ln-dependent homolog, XoxF (10). While MxaFI and XoxF have similar active sites, crystal structures confirmed that the XoxF active site contains an extra aspartic acid residue that is essential for Ln binding (11). Further studies found that Ln-dependent enzymes are not isolated to methylotrophic bacteria nor to exhibiting methanol dehydrogenase activity; the ethanol dehydrogenase ExaF from *M. extorquens* AM1 as well as the ethanol dehydrogenase PedH from *Pseudomonas* *putida* KT2440 were recently discovered to be Ln-dependent (12, 13).

All known Ln-dependent enzymes to date are periplasmic proteins, but the details of Ln acquisition, transport, and integration into enzymes are not fully understood. The Ln utilization and transport (*lut*) cluster that was discovered in *M. extorquens* AM1 (14) and the closely related *M. extorquens* PA1 is required for Ln-dependent growth (15). In this system, Ln are initially brought into the periplasm through the TonB-dependent receptor LutH. There is currently no experimental evidence of free Ln in the periplasm. Rather, Ln are then transported to the cytoplasm via the *lut*-encoded ABC transport system, but the necessity of Ln reaching the cytoplasm is not yet understood. While it seems that the *lut* cluster encodes the primary system for Ln uptake, it remains unknown whether other transport systems are also used.

The presence of a Ln-chelating metallophore has long been proposed (15, 16). Methylotrophs like *M. extorquens* AM1 are known to thrive in mesophilic environments that are rich in poorly soluble Ln such as the soil and the phyllosphere (the arial region of plants) where a mechanism for solubilization would be necessary for Ln-dependent methylotrophic growth. Concentrations of Ln in the phyllosphere range from 0.7 μg/g dry weight to 7 μg/g dry weight, but bioavailable Ln concentrations are likely far less (15, 17). In most soil environments, Ln are found in highly insoluble oxide and phosphate forms, usually in minerals and ores such as monazite and bastnäsite (18). Despite this, most work done in a laboratory setting has used soluble chloride salts to understand Ln biology.

In this study, we utilize Ln sources of high (NdCl_3_) and low (Nd_2_O_3_) solubility to identify and structurally characterize a metallophore involved in Ln metabolism, a lanthanophore we have named methylolanthanin (MLL, **1**). We identified the *mll* BGC through assessment of *M. extorquens* AM1’s transcriptional response to a low solubility Ln source. We find overexpression of the MLL biosynthetic genes *in trans* increases growth and Ln bioaccumulation and adsorption, while deletion of these genes generates a severe defect in Ln bioaccumulation and adsorption. Furthermore, we demonstrate the Ln-binding of MLL and find that exogenous addition of the compound to a mutant lacking the capability to biosynthesize it improves growth yield. Finally, we demonstrate that the *mll* gene cluster is differentially responsive to iron limitation dependent upon the presence of lanthanides and *mxaF*.

## Results

### Unbiased Identification of Ln Solubility Gene Networks.

**Fig. 1.**
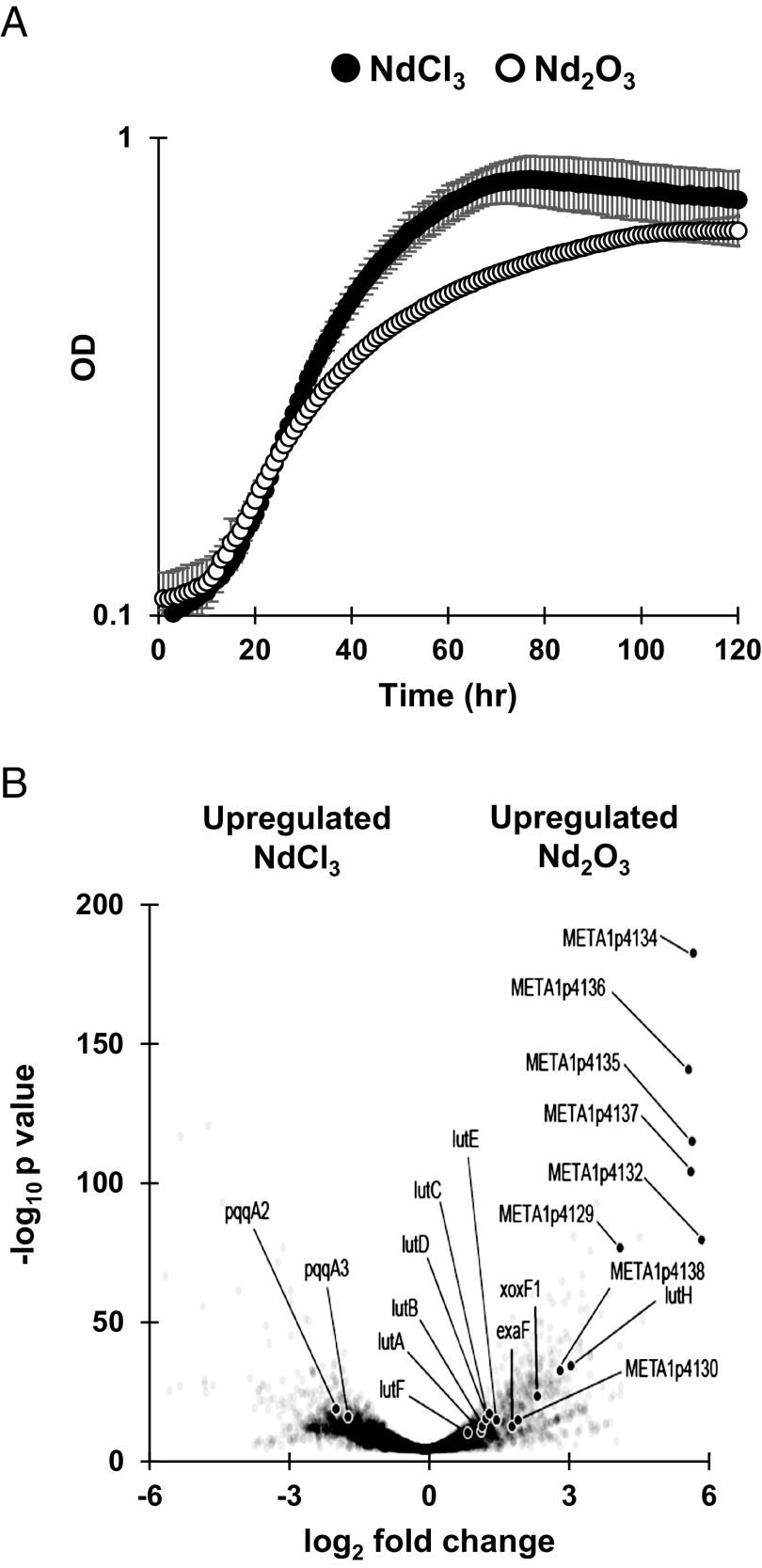
Differences in Ln source solubility affect growth and transcriptional response of *M. extorquens*. (*A*) Growth rate of Δ*mxaF* is significantly increased during growth on soluble NdCl_3_ (filled circles) compared to poorly soluble Nd_2_O_3_ (open circles). Individual data points represent the mean of three replicates and error bars represent the SD. (*B*) Volcano plot of DEGs comparing NdCl_3_ and Nd_2_O_3_ conditions with genes for methylolanthanin, META1p4132 through META1p4138, highlighted. Individual data points represent the mean of three replicates.

To better understand the impacts of Ln solubility on methylotrophy in *M. extorquens* AM1, we measured growth with methanol as an energy source with soluble NdCl_3_ and poorly soluble Nd_2_O_3_ using Δ*mxaF*, a strain that lacks catalytically active MxaFI and is therefore dependent on Ln for growth on methanol (19). Higher Ln solubility resulted in significantly faster growth (*P* value = 0.001) (Fig. 1*A*). We employed RNA-seq transcriptomics to gain unbiased insight into the reasons for this significant growth defect with Nd_2_O_3_. We identified 1,468 differentially expressed genes between the NdCl_3_ and Nd_2_O_3_ conditions (Fig. 1*B*).

When Nd_2_O_3_ was provided, the gene encoding the primary Ln-dependent MDH (*xoxF1*) was among the most up-regulated genes, exhibiting 5-fold upregulation when compared to the soluble Ln condition (Fig. 1*B*). *xoxF1* was up-regulated in both Ln conditions compared to a wild-type control with no lanthanides (NCBI GEO Accession No. GES244060) (20). This result corroborates previous findings on the upregulation of *xox* genes generated by the presence of exogenous LaCl_3_ (21–24). We also found the gene encoding the Ln-dependent ethanol dehydrogenase *exaF* was shown to have a threefold increase in expression during growth with Nd_2_O_3_ compared to growth with NdCl_3_, suggesting that alternative Ln alcohol dehydrogenases are important when Ln are less bioavailable.

Previous studies have shown that although upregulation of *xoxF1* occurs as a response to soluble Ln, concomitant upregulation of the PQQ biosynthetic genes does not occur (21, 23). We find similar results in this study, as most of the genes encoding the PQQ biosynthetic machinery were not significantly up-regulated in the NdCl_3_ condition. Interestingly, the orphan *pqqA* copies *pqqA2* and *pqqA3* were up-regulated fourfold in the NdCl_3_ condition compared to the Nd_2_O_3_ condition (Fig. 1*B*).

The *lut* cluster, which encodes the TonB-dependent transporter LutH, an ABC transport system, and various periplasmic proteins, is essential for light Ln transport. Previous work has shown expression of *lutH* does not change in response to soluble LaCl_3_ (21). In this study, *lutH* was down-regulated ninefold in the Δ*mxaF* NdCl_3_ condition compared to the WT no Ln condition and up-regulated ninefold compared to the Δ*mxaF* Nd_2_O_3_ condition, denoting tight regulation of the TonB-dependent transporter based on Ln species (Fig. 1*B*). The remaining *lut* genes exhibited 2-fold upregulation on average during growth with Nd_2_O_3_.

### Identification of a Putative Lanthanophore Locus.

**Fig. 2.**
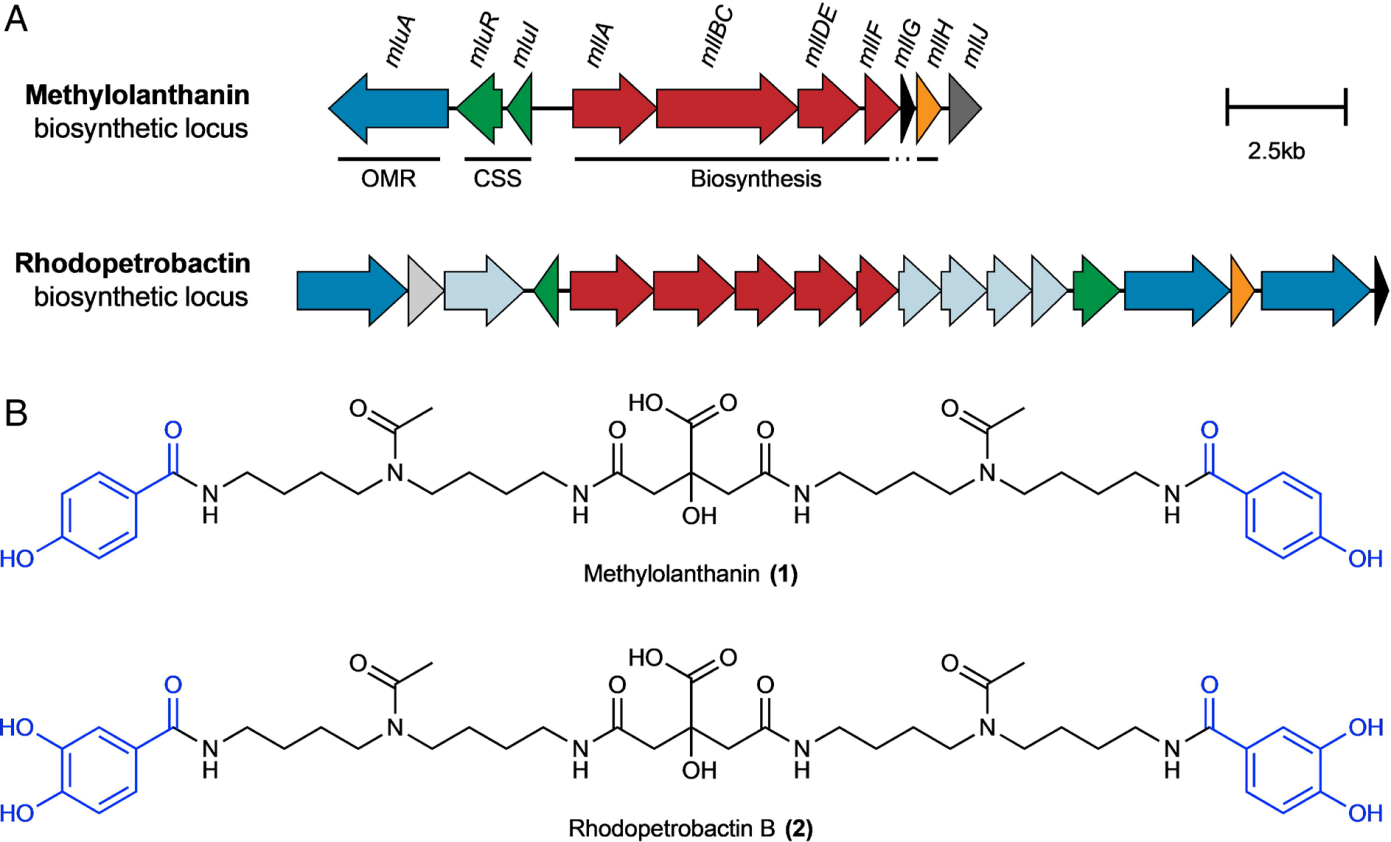
BGCs and chemical structures of methylolanthanin (**1**) and the related siderophore rhodopetrobactin (**2**). *A*, The methylolanthanin BGC from *M. extorquens* AM1 (*mll/mlu*, META1p4129-4138) and the rhodopetrobactin BGC from *Rhodopseudomonas palustris* TIE-1 (25). Homologous pathways between BGCs share the same color. Genes are drawn to scale. OMR, TonB-dependent outer membrane receptor; CSS, cell-surface signaling. Additional homologous clusters are presented in *SI Appendix*, Fig. S1. (*B*) Chemical structures of methylolanthanin (**1**) and rhodopetrobactin (**2**), which differ by the presence of 4-HB vs. 3,4-DHB, respectively (in blue).

Of all genes identified in RNAseq analysis, the genes META1p4129 through META1p4138 were the most highly up-regulated in the Nd_2_O_3_ condition, with an average increase in expression of 32-fold compared to growth with NdCl_3_ (Fig. 1*B* and Table 1). The locus, which we have named *mll* (for methylolanthanin, vide infra), is homologous to BGCs responsible for the transport, regulation, and biosynthesis of NRPS-independent siderophores containing citrate and 3,4-dihydroxybenzoate (3,4-DHB) chelating groups, including rhodopetrobactin, petrobactin, and roseobactin (Fig. 2 and *SI Appendix*, Fig. S1) (25). META1p4132-4135 (*mllA, mllBC, mllDE*, and *mllF*) are homologous to the well-studied petrobactin locus in *B. subtilis*, *asbABCDEF* (26), differing by two sets of gene fusions; the fusion between *asbD* and *asbE* homologs is also present in the rhodopetrobactin and roseobactin loci, while the *mllBC* fusion has not been observed in characterized homologs. The presence of putative acetyltransferase *mllH* (META1p4137) suggested the incorporation of an acetylated (homo)spermidine linker, as seen in rhodopetrobactin. META1p4129-4131 (*mluARI*, **m**ethylo**l**anthanin **u**ptake) putatively encode, respectively, a TonB-dependent outer membrane receptor, an anti-sigma factor, and a sigma factor. Homologous proteins form a cell-surface signaling pathway, where the import of a ferric siderophore induces further expression of the receptor (27). Two domains of unknown function are encoded in the cluster. META1p4136 (*mllG*) belongs to DUF2218 and is also present in the rhodopetrobactin locus. Homologous VCA0233 from *Vibrio cholerae* neighbors an iron uptake regulator and xeno-siderophore uptake genes (28), suggesting DUF2218 is involved in regulation or transport rather than biosynthesis. META1p4138 (*mllJ*) belongs to ferritin-like DUF4142 and is putatively exported into the periplasm. A phylogenetic tree of representative *Methylorubrum* and *Methylobacterium* genomes was reconstructed using PhyloPhlAn, and ITOL was used to map the presence of homologous BGCs, as determined by a custom version of antiSMASH (29–31). The majority of *Methylorubrum* species, forming a single clade, contain homologs of the *mll* locus, as well as *Methylobacterium currus* TP3 and *Methylobacterium aquaticum* BG2 (*SI Appendix*, Fig. S1). The *mll* locus was not found in any of the more distantly related 85 genomes previously found to contain XoxF homologs (32). Together, the insights that the *mll* locus is 1) up-regulated during growth with an insoluble Ln source, 2) contains genes commonly found in metallophore BGCs, and 3) is conserved across many *Methylorubrum/Methylobacterium* strains, all suggested that the cluster produces a rhodopetrobactin-like lanthanophore.

**Table 1.** Comparison of methylolanthanin and rhodopetrobactin BGCs

Gene	Locus tag	log2 fold change	q-value	Putative product	Rhodopetrobactin BGC homolog	% ID
*mluA*	META1p4129	4.2	3.70E-84	TonB-dependent receptor	Rpa1_2620	62
*mluR*	META1p4130	2	3.00E-12	Anti-sigma factor	Rpa1_2623	30
*mluI*	META1p4131	6.1	3.60E-58	Sigma factor	Rpa1_2633	30
*mllA*	META1p4132	6	1.20E-87	*asbA*-like NIS synthetase	Rpa1_2632	50
*mllBC*	META1p4133	6.3	1.10E-165	*asbB*-like NIS synthetase and *asbC*-like adenylation domain	Rpa1_2631 Rpa1_2630	52 56
*mllDE*	META1p4134	5.8	3.60E-209	*asbD*-like aryl carrier protein and *asbE*-like ligase fusion	Rpa1_2629	54
*mllF*	META1p4135	5.8	8.30E-130	*asbF*-like 3,4-DHB synthase	Rpa1_2628	52
*mllG*	META1p4136	5.7	3.20E-160	DUF2218 family protein	Rpa1_2619	67
*mllH*	META1p4137	5.8	6.80E-117	GNAT family acetyl transferase	Rpa1_2621	74
*mllJ*	META1p4138	2.9	6.10E-33	DUF4142 family protein	-	

### Structural and Functional Elucidation of Methylolanthanin.

**Fig. 3.**
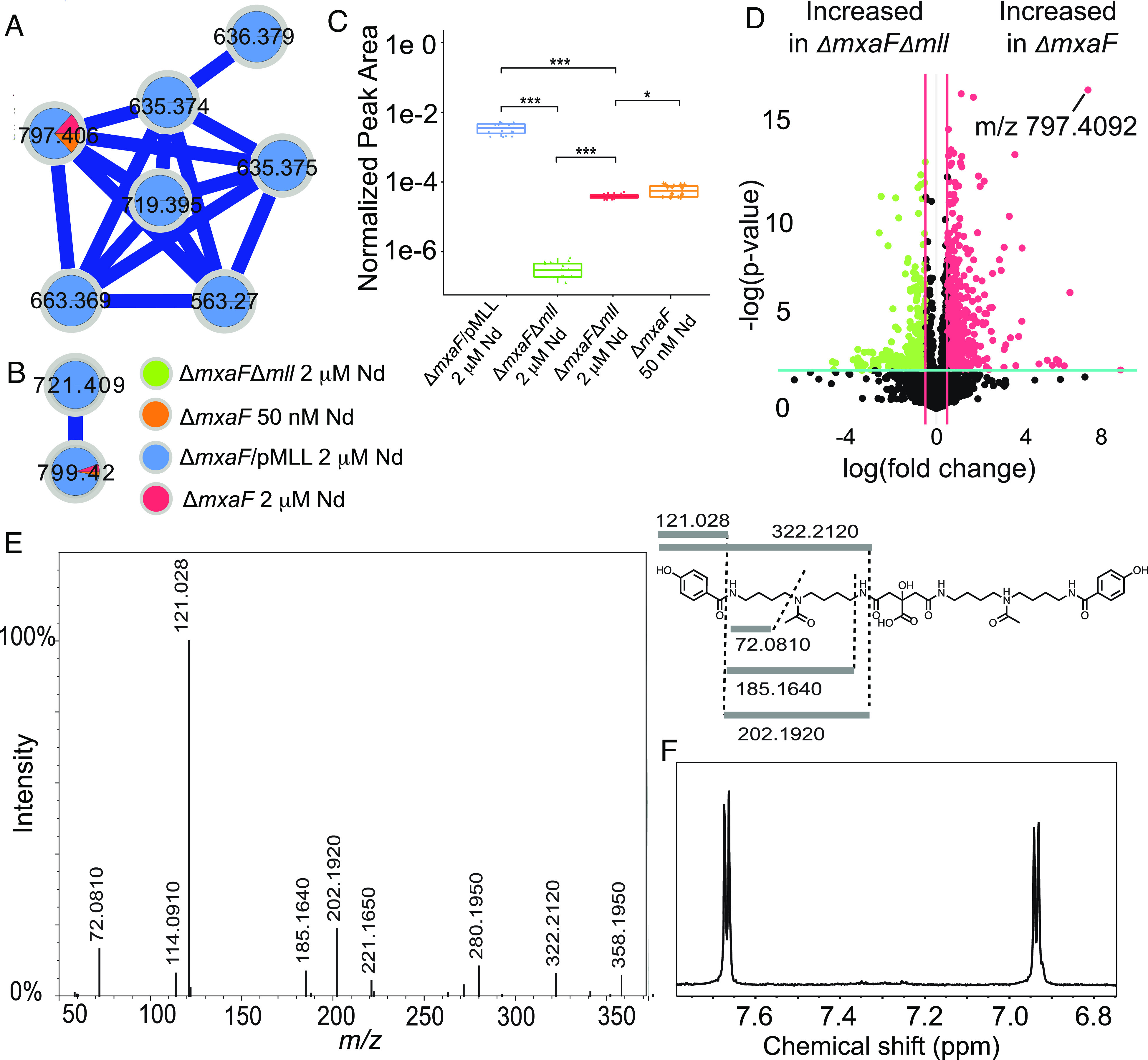
Identification of methylolanthanin via molecular networking and MS/MS. (*A*) ESI-UHPLC–MS/MS in negative ionization mode reveals a molecular family of MS/MS spectra only found in Δ*mxaF* (orange and red) and Δ*mxaF*/pMLL (blue) but not in Δ*mxaF*Δ*mll* (green). (*B*) ESI-UHPLC-MS/MS in positive ionization mode reveals a molecular family of MS/MS spectra only found in *ΔmxaF* (orange and red) and *ΔmxaF*/pMLL (blue) but not in Δ*mxaF*Δ*mll* (green). Two features (*m/z* 799.4232 in positive mode/*m/z* 797.4092 in negative mode and *m/z* 721.4114 in positive mode/*m/z* 719.3978 in negative mode) are found in both positive and negative ionization mode molecular families. (*C*) Boxplots of the normalized peak area of feature *m/z* 797.4092 in negative mode reveal significant differences between peak area in all conditions. Statistics were calculated with n = 20 per group Kruskal–Wallis, followed by pairwise Wilcoxon tests and Benjamini–Hochberg (BH) correction (**P < 0.001, *P < 0.05). Upper and lower whiskers extend to the closest value to ±1.5 * IQR. (*D*) *m/z* 797.4092 is one of the most significantly increased features in Δ*mxaF* vs. Δ*mxaF*Δ*mll* supernatants when analyzed using ESI-UHPLC-MS/MS in positive ionization mode. Volcano plot analysis was performed with n = 5 per group. Fold changes 0.5 and a *P*-value < 0.01 are highlighted. (*E*) The consensus MS/MS spectrum in positive mode is consistent with the proposed structure, as highlighted by the key fragments in the chemical structure. (*F*) The NMR aromatic region of isolated methylolanthanin supports a parasubstituted monohydroxybenzoate moiety (^1^H NMR spectrum, 800 MHz, D_2_O + 0.003% TMSP).

To determine the identity of the putative lanthanophore produced by the *mll* cluster, mutants lacking (Δ*mxaF*Δ*mll*) and overexpressing (Δ*mxaF*/pMLL) the *mll* BGC *(mllA* through *mllJ*) were constructed. The genome of the *mll* deletion strain was sequenced, and it was confirmed that no additional mutations had been acquired. Supernatant extracts were analyzed using ultra-high-performance liquid chromatography–tandem mass spectrometry (UHPLC–MS/MS) in both positive and negative ionization modes. Classical molecular networking using the Global Natural Products Social (GNPS) Molecular Networking platform (33) revealed only one molecular family that was found in Δ*mxaF* supernatants and not in Δ*mxaF*Δ*mll* supernatants, containing a compound with a mass to charge ratio (*m/z*) of 799.4232 in positive mode and 797.4092 in negative mode (Fig. 3 *A* and *B* and *SI Appendix*, Fig. S2). Multivariate and univariate (Fig. 3 *C* and *D* and *SI Appendix*, Fig. S2) statistical analyses were performed in parallel on blank subtracted, normalized, and imputed feature tables processed after feature finding in MZmine 3 (34). Volcano plot analysis comparing *ΔmxaF* supernatant with Δ*mxaF*Δ*mll* supernatant was consistent with classical molecular networking results, revealing that the putative *mll* product (positive mode *m/z* 799.4232; negative mode *m/z* 797.4092) illustrates the second-highest fold-change and most significant *P*-value in negative mode data (Fig. 3*D*), and the putative *mll* product is differentially abundant across Δ*mxaF*, *mll* knockout, and *mll* overexpression samples.

Tandem mass spectrometry (MS/MS) data collected in both negative and positive modes revealed fragments that fit with a proposed structure that resembles rhodopetrobactin B minus two oxygen atoms. Key fragments in positive mode consensus MS/MS include *m/z* 322.2120, 202.1920, 185.1640, 121.0280, and 72.0810 (Fig. 3*E*). These fragments indicate the presence of acetylated homospermidine (4,4′-diaminodibutylamine) and monosubstituted hydroxybenzoic acid (HB). The presence of two HB moieties accounts for the mass difference with rhodopetrobactin B, which instead contains two 3,4-dihydroxybenzoic acid (3,4-DHB) moieties. The neutral loss of 156.006 (between fragments at *m/z* 299.1590 and 143.1540) is consistent with the presence of citrate.

The molecule was isolated to confirm the proposed structure and determine the position of the hydroxyl group in the HB. NMR spectroscopy of the purified compound revealed two doublets in the aromatic region, clearly showing the *para* substitution of the HB moiety (Fig. 3*F*). Thus, the structure of the molecule, which we named methylolanthanin (MLL, **1**), was fully established and confirmed by NMR (*SI Appendix*, Fig. S3): a central citrate group is linked to two 4-HB moieties via homospermidine residues, each of which is acetylated at the central amine (Fig. 2*B*). Signals in the ^1^H NMR spectrum show varying degrees of unexpected splitting that can be explained by the presence of four conformers of roughly equal abundance stemming from different orientations of the two acetyl groups (*SI Appendix*, Fig. S4). The diastereotopic methylene groups in the citrate moiety show ^1^H NMR signals for four overlapping AB systems instead of two doublets with a roof effect (*SI Appendix*, Figs. S3 and S4). The overlap of the signals depends on the solvent system used (H_2_O/D_2_O vs. D_2_O; *SI Appendix*, Fig. S4). The signals of the secondary amine protons (only observed in H_2_O/D_2_O) also showed additional splitting most probably caused by the four possible conformers (see *SI Appendix*, Fig. S4 for deconvoluted signals).

**Fig. 4.**
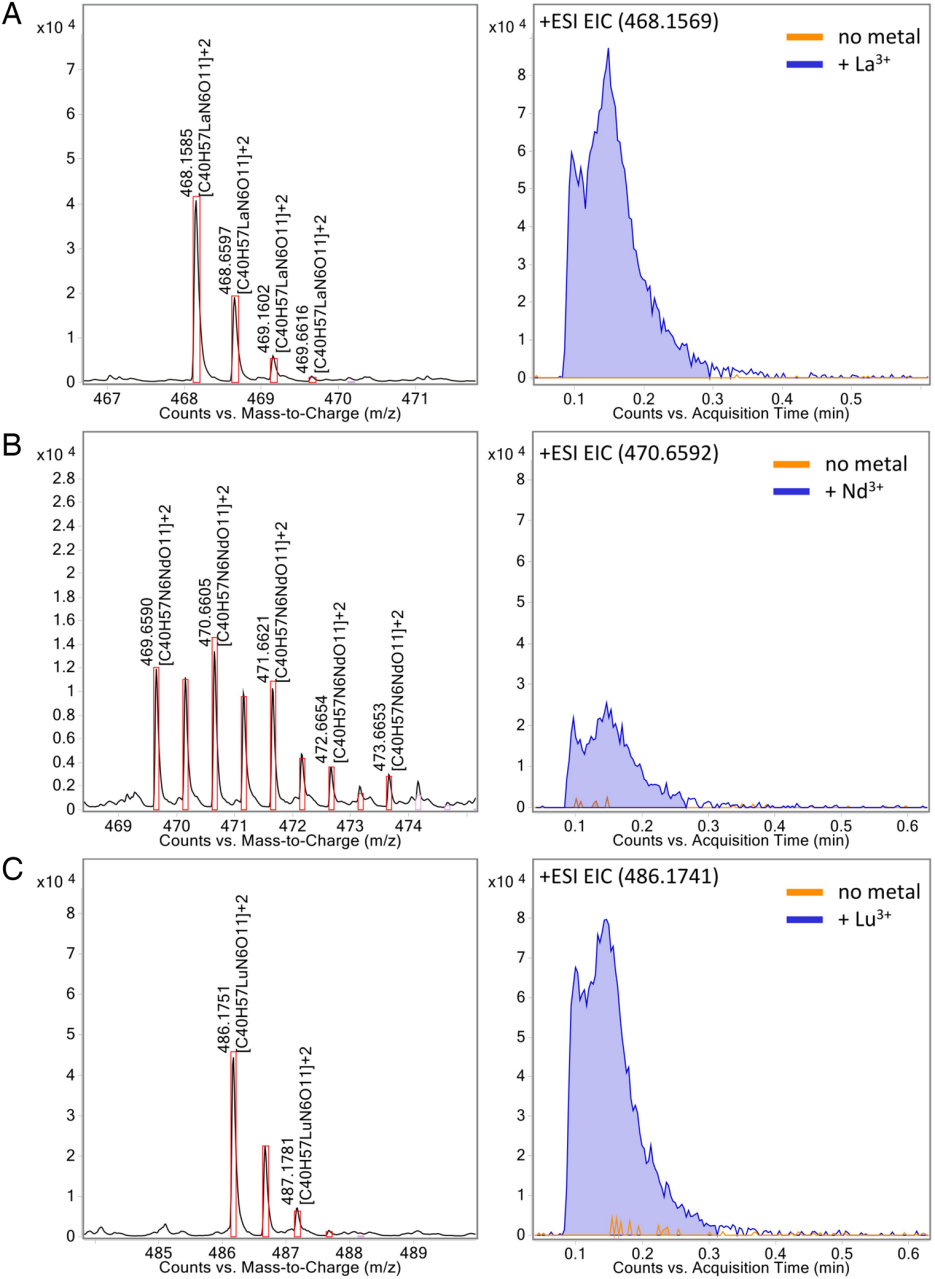
Methylolanthanin binds lanthanum (La), neodymium (Nd), and lutetium (Lu). EICs of methylolanthanin-Ln^3+^ complexes and corresponding mass spectra showing the isotopic pattern supporting complex formation. (*A*) La^3+^, (*B*) Nd^3+^, and (*C*) Lu^3+^. Theoretical isotopic patterns are indicated by boxes.

Furthermore, we were able to show via direct injection mass spectrometry that methylolanthanin is capable of binding lanthanides of varying sizes by injecting premixed samples of MLL with La(III), Nd(III), and Lu(III). In each case, a complex of the formula [MLL-H^+^+Ln^3+^]^2+^ can be found with the corresponding isotopic pattern of La, Nd, and Lu (Fig. 4).

### Phenotypic Analysis of Methylolanthanin Mutants.

**Fig. 5.**
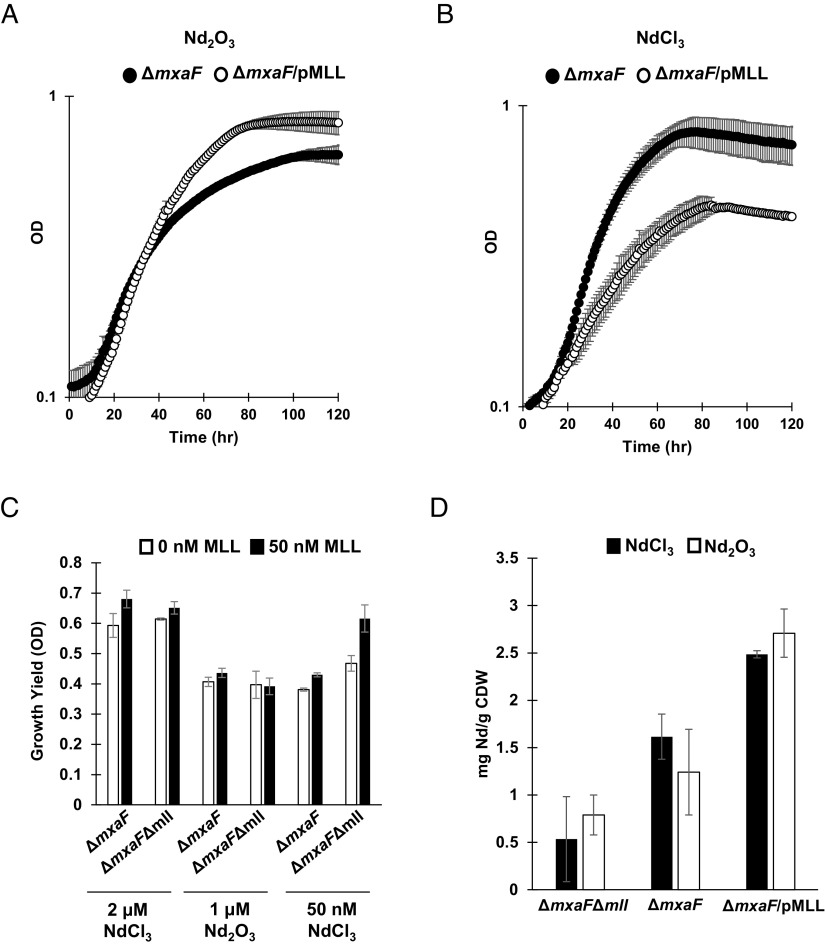
Manipulation of *mll* affects growth yield and neodymium bioaccumulation. (*A*) Growth of Δ*mxaF* (filled circles) and Δ*mxaF*/pMLL (empty circles) with 1 μm Nd_2_O_3_. Individual data points represent the mean of three replicates and error bars represent SD. (*B*) Growth of Δ*mxaF* (filled circles) and Δ*mxaF*/pMLL (empty circles) with 2 μm NdCl_3_. Individual data points represent the mean of three replicates and error bars represent SD. (*C*) Growth yield of Δ*mxaF* and Δ*mxaF*Δ*mll* growing with 2 μm NdCl_3_, 1 μm Nd_2_O_3_, or 50 nM NdCl_3_, with or without 50 nM MLL. Values represent the mean of three replicates and error bars represent SD. (*D*) Intracellular accumulation of Nd by Δ*mxaF*Δ*mll*, Δ*mxaF*, and Δ*mxaF*/pMLL with 2 μm NdCl_3_ and 1 μm Nd_2_O_3_. Values represent the mean of three replicates and error bars represent SD.

To better understand the effect methylolanthanin has on the physiology of *M. extorquens* AM1, the growth and Ln bioaccumulation and adsorption of Δ*mxaF*Δ*mll* and Δ*mxaF*/pMLL were studied alongside a Δ*mxaF* control. As shown above, Δ*mxaF* exhibited a clear defect when grown with Nd_2_O_3_ compared to when grown with NdCl_3_ (Fig. 1*A*). This defect was rescued when the *mll* cluster was overexpressed; Δ*mxaF*/pMLL Nd_2_O_3_ cultures grew at a rate of 0.026 h^−1^, a nearly 50% increase compared to the growth of Δ*mxaF* with Nd_2_O_3_ and a growth rate closer to that of Δ*mxaF* on NdCl_3_ (0.037 h^−1^) (Fig. 5*A*). Interestingly, Δ*mxaF*/pMLL cultures incurred a defect in growth rate when compared to Δ*mxaF* when grown with soluble NdCl_3_ (Fig. 5*B*). Pure methylolanthanin (50 nM), isolated from Δ*mxaF*/pMLL cultures, was added to Δ*mxaF* or Δ*mxaF*Δ*mll* growing with 2 μm NdCl_3_, 50 nM NdCl_3_, or 1 μm Nd_2_O_3_ to assess the effect of the molecule on growth. Presence of exogenous methylolanthanin significantly increased growth yield (maximal OD in stationary phase) of both cultures when grown with 2 μm NdCl_3_ (*P* values = 0.036 and 0.037) or 50 nM NdCl_3_ (*P* values = 0.0158 and 0.00592) (Fig. 5*C* and *SI Appendix*, Fig. S5).

As Ln-dependent growth of *M. extorquens* AM1 has previously been shown to be tied to Ln bioaccumulation, inductively coupled plasma mass spectrometry (ICP-MS) was used to measure cellular concentrations of Nd within Δ*mxaF,* Δ*mxaF*Δ*mll*, and Δ*mxaF*/pMLL grown with Nd_2_O_3_ or NdCl_3_ (14, 35). Δ*mxaF* bioaccumulated and adsorbed Nd to levels previously reported for other light Ln (Fig. 5*D*) (35). Deletion of *mll* decreased Nd bioaccumulation and adsorption with both Ln sources, notably by 1.8-fold in the NdCl_3_ condition, while overexpression of *mll* increased Nd bioaccumulation and adsorption by 3.5-fold on average (Fig. 5*D*).

### *mll* Promoter Responds to Neodymium and Iron.

**Fig. 6.**
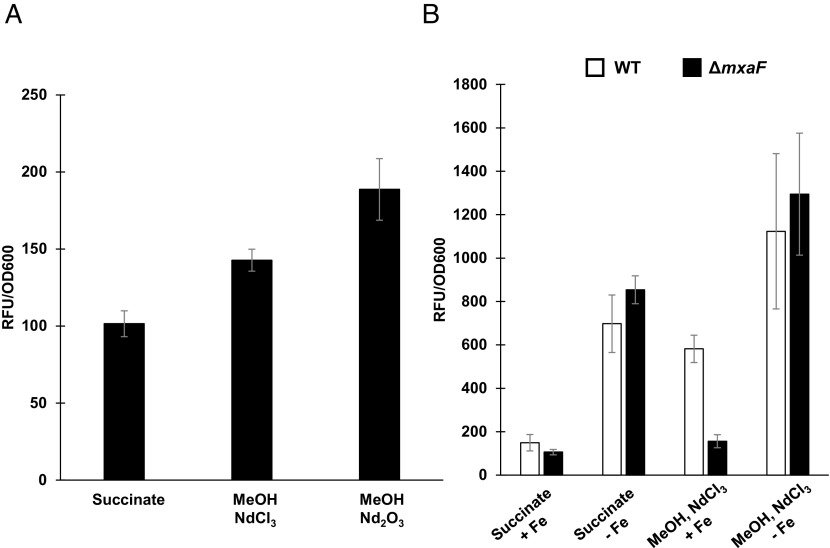
*mll* transcription is responsive to neodymium and iron. (*A*) Fluorescence of *mll* promoter fusion strain, Δ*mxaF*/pAZ6 in response to neodymium addition and neodymium solubility. Bars represent the mean of three replicates and error bars represent SD. (*B*) Fluorescence of *mll* promoter fusion strains, Δ*mxaF*/pAZ6 (black) and WT/pAZ6 (white), in response to iron limitation. Bars represent the mean of three replicates and error bars represent SD. All values have had background fluorescence of an empty vector control subtracted.

As methylolanthanin has shared structural similarities to the siderophore rhodopetrobactin, the response of its production to iron limitation was measured through a fluorescent promoter fusion assay. The intergenic, 700 bp region upstream of the first *mll* biosynthetic gene, *mllA*, was fused to mCherry to generate pAZ6 (*SI Appendix*, Fig. S6). In a Δ*mxaF* background, activity of the *mll* promoter increased when cells were provided methanol and NdCl_3_ compared to multicarbon growth on succinate without lanthanides. Activity of the *mll* promoter further increased when the cells were provided methanol and Nd_2_O_3_, a result that mirrors *mll* expression levels determined by RNAseq (Figs. 1*B* and 6*A*).

WT/pAZ6 and Δ*mxaF*/pAZ6 were each grown in iron-limiting medium and the activity of the *mll* promoter was measured. Both strains showed increases in promoter activity in response to iron limitation, regardless of carbon source. Promoter activity in response to iron-deplete medium was the same between strains when grown in succinate (*P* = 0.193) or with methanol and NdCl_3_ (*P* = 0.376). Promoter activity in response to iron-replete medium was also the same between strains when grown in succinate (*P* = 0.152). The only condition in which the promoter activity differed between the strains was when grown in iron-replete medium with methanol and NdCl_3_, where activity was significantly lower for Δ*mxaF*/pAZ6 compared to WT/pAZ6 (*P* = 0.0014) (Fig. 6*B*).

## Discussion

Metallophores for iron, zinc, copper, nickel, and molybdenum have been chemically and biosynthetically elucidated. No Ln-specific metallophore (lanthanophore) has been discovered even as Lns are essential cofactors for certain alcohol dehydrogenases (36). Here, we report the chemical structure, biosynthesis, and physiological relevance for methylolanthanin (**1**), a Ln-binding metallophore involved in lanthanide metabolism. The narrow taxonomic distribution of *mll* (*SI Appendix*, Fig. S1) and the common function of homologous molecules as Fe-binding siderophores suggest that the methylolanthanin pathway entered *Methylorubrum* through horizontal gene transfer and evolved to chelate Ln in addition to Fe, although further study is required to confirm this hypothesis. It has been previously shown by Klebensberger and coworkers that growth of the Ln-utilizer *P. putida* KT2440 in the presence of lanthanides is influenced by iron levels in the medium (37). It is likely that Ln and Fe metabolism are strongly intertwined; here we see that *mll* promoter activity is responsive to both neodymium and iron (Fig. 6). Interestingly, we find the activity of the *mll* promoter to perturbations in Nd and Fe levels to be variable depending on the presence or absence of *mxaF*, suggesting a complex relationship between proteins involved in the Ln-switch, lanthanides, and iron.

Metallophores benefit the organisms that produce them and the “cheaters” that utilize them (38, 39). Methylolanthanin may provide a benefit for producers in environments where Ln are poorly bioavailable, such as the soil and phyllosphere, whereas Ln-utilizing extremophiles in Ln-richly-bioavailable environments would not require the production of such a molecule. Surveying the RefSeq nonredundant protein record for organisms that encode clusters like the *mll* cluster, we find similar clusters in mesophilic, methylotrophic Ln-utilizing organisms but not in extremophilic, methylotrophic Ln-utilizing organisms (ex: *Methylacidiphilum fumariolicum* SolV, isolated from a volcanic mudpot) (*SI Appendix*, Fig. S1).

Growth of Δ*mxaF*Δ*mll* (*SI Appendix*, Fig. S5) denotes that production of methylolanthanin is not essential under the conditions tested. This leaves open questions about alternative systems for Ln uptake, perhaps through nonspecific ion transporters or through the production of other chelation systems. Previous work has shown extracts of a *M. extorquens* strain evolved to use the heavy Ln Gd have exhibited a sixfold increase in PQQ levels compared to Δ*mxaF* extracts (35). Additionally, PQQ has been shown to be able to bind Ln (40, 41). While in this study we did not observe an increase in the expression of most PQQ biosynthetic genes during growth with poorly bioavailable Nd_2_O_3_, we did observe increased expression of two orphan *pqqA* genes (Fig. 1*B*). *pqqA*, encoding the peptide precursor to PQQ, has previously been reported to be nonessential for PQQ biosynthesis in *M. extorquens* AM1, but orphan *pqqA* copies were not investigated in these studies (42). In *Methylovorus* sp. MP688, which is equipped with five *pqqA* copies, the availability of PqqA has been shown to be a rate-determining step in PQQ biosynthesis (43). Taken together, it is possible that PqqA could be a component of an alternative Ln uptake system.

It is known that *M. extorquens* AM1 is responsive to Ln levels as low as 2.5 nM, but it is not yet known why Ln are bioaccumulated at much higher levels (19, 44). *M. extorquens* AM1 is a candidate to recover lanthanides selectively from electronic waste and old magnets (44). Previously it has been shown that *M. extorquens* AM1 can selectively bioaccumulate Nd from NdFeB magnets, hence neodymium salts were chosen in this study. Clearly, *M. extorquens* AM1 stores far more Ln than it requires for robust growth. Cultures of Δ*mxaF*Δ*mll* exhibit a 30% decrease in bioaccumulation and adsorption yet can grow similarly to Δ*mxaF* cultures that exhibit typical levels of Ln bioaccumulation and adsorption (Fig. 5*D* and *SI Appendix*, Fig. S5). Further research is needed to understand the complex relationship between growth and Ln storage.

The most striking feature of methylolanthanin is the 4-HB moiety, which has not been reported in any characterized metallophore. Baars et al., reported small amounts of rhodopetrobactin analogs from *R. palustris*, rhodopetrobactins A - O, A - 2O, and B - O, that also contain one or two HB moieties (possibly 4-HB) in place of 3,4-DHB. The molecular weight of methylolanthanin would be equivalent to “rhodopetrobactin B - 2O”, which was not observed by Baars et al., Conversely, we did not observe any putative 3,4-DHB–containing analogues in the *M. extorquens* AM1 supernatant. In petrobactin biosynthesis, 3,4-DHB is produced directly from dehydroshikimate by the dehydratase AsbF (45), and thus 4-HB and methylolanthanin are likely bona fide analogs of 3,4-DHB and rhodopetrobactin B rather than their biosynthetic intermediates. The presence of 4-HB in a metallophore is unexpected, as most metallophore structures contain bidentate chelating moieties. We have shown elsewhere that Fe bioaccumulation and adsorption remain unaffected by overexpression of *mll* (44). The Ln affinity of methylolanthanin may be stalled at a local fitness peak, where evolving greater affinity for Ln binding would incur a fitness disadvantage that cannot occur under selection. Siderophores, zincophores, and other metallophores exhibit convergent evolution, where biosynthetically unrelated molecules share the same function. The existence of methylolanthanin suggests that other lanthanophores may have evolved, possibly with varying lanthanide affinity and selectivity.

The identification of methylolanthanin, a structurally unique lanthanide metallophore, indicates that lanthanides can interact with microbial specialized metabolite production. Here, we have shown not only the structure of this molecule but also its physiological relevance; methylolanthanin production is highly up-regulated in response to poorly soluble lanthanides and is required for wild-type levels of lanthanide bioaccumulation and adsorption. We also find that *M. extorquens* AM1 can be engineered to bioaccumulate and adsorb high levels of lanthanides through overproducing methylolanthanin, an important finding for the use of this organism in biomining (44). Looking forward, we anticipate more lanthanophores to be identified alongside the mechanisms for their production, regulation, and transportation.

## Materials and Methods

### General Working and Culturing Conditions.

MilliQ grade water and HPLC grade solvents were used in this work, unless otherwise stated. For mass spectrometry and related experiments, LCMS grade solvents were used. Culturing occurred under sterile conditions. *M. extorquens* AM1 strains [WT Rif^R^ derivative (46), Δ*mxaF* (47), Δ*mxaF*/pMLL, and Δ*mxaF*Δ*mll*] were grown in Hypho medium (48) (K_2_HPO_4_ 14.5 mM, NaH_2_PO_4_ 18.8 mM, MgSO_4_·7H_2_O 0.8 mM, (NH_4_)_2_SO_4_ 3.8 mM, FeSO_4_·7H_2_O 3.69 μm, Na_2_·EDTA·2H_2_O 26.86 μm, CaCl_2_·2H_2_O 9.98 μm, MnCl_2_·4H_2_O 5.11 μm, (NH_4_)_6_Mo_7_O_2_ 0.18 μm, CuSO_4_ 1.26 μm, CoCl_2_·6H_2_O 6.72 μm, ZnSO_4_·7H_2_O 15.3 μm) but reducing the phosphate concentration by half, from 33.3 mM to 16.6 mM (referred to as ½ Hypho). FeSO_4_·7H_2_O was omitted for iron-deplete media. 3 mL cultures with 15 mM succinate (MilliporeSigma) were grown overnight in 15 mL round-bottom glass culture tubes (Thermo Fisher Scientific) at 30 °C and 200 rpm and then diluted into the desired volume of fresh ½ Hypho to an OD600 of 0.1. When necessary, sucrose, kanamycin, chloramphenicol, ampicillin, and methylolanthanin were added to final concentrations of 5%, 50 μg/mL, 12.5 μg/mL, 50 μg/mL, and 50 nM, respectively. Growth curves were obtained with 650 µL cultures grown in transparent 48-well plates (Corning) incubated at 30 °C and 548 rpm using a Synergy HTX plate reader (Biotek). Excitation and emission spectra used for promoter fusion assays were 585 nm and 620 nm, respectively. Fluorescence units were divided by OD600 at stationary phase for both test conditions and for an empty, uninduced pTE1877 control. The above conditions were used unless otherwise noted.

### RNA sequencing.

50 mL ½ Hypho medium supplemented with 50 mM methanol and 1 μm Nd_2_O_3_ (99.99%, Chempur) or 2 μm NdCl_3_ (99.99%, MilliporeSigma) was used to culture WT AM1 and Δ*mxaF* in 250 mL glass Erlenmeyer flasks to mid-exponential phase (OD600 = 0.6). Total RNA samples were generated and quality was corroborated as described previously (49). rRNA depletion using the Ribo-Zero RNA Plus rRNA Depletion Kit (Illumina), library preparation, and Illumina Hi-Seq sequencing were performed by the Microbial Genome Sequencing Center (MiGS). Using KBase (50), reads were aligned with HISTAT2, transcripts were assembled with StringTie, and DEGs were identified using DESeq2.

### Comparative Genomics.

Gene cluster functions were predicted using hmmscan (EMBL webserver) (51, 52) and through NCBI BLAST (53) matches against the rhodopetrobactin BGC from *R. palustris* TIE-1 (25). Genomes were retrieved from NCBI RefSeq (54) in Genbank and protein fasta formats using ncbi-genome-download v0.3.3 (55) for phylogenetic analysis. The following genomes were selected: complete genomes from *Methylorubrum* and *Methylobacterium*, draft representative genomes from *Methylorubrum*, and three additional *Methylobacteriaceae* genomes as an outgroup (*SI Appendix*, Fig. S1 and Table S1). The phylogenetic tree was reconstructed from conserved protein groups using PhyloPhlAn v3.0.67 with the following configuration parameters: “-d a --msa mafft --tree1 raxml --db_aa diamond --map_aa diamond” and “--diversity medium” (56–58). Homologs of the methylolanthanin locus were found using a custom version of antiSMASH v7.0.1 (31) (https://doi.org/10.5281/zenodo.8348225), detection rule for homologs of the petrobactin biosynthetic genes *asbABCDE*. A BGC region met the “AsbABCDE” rule if it contained matches to existing antiSMASH models for IucA_IucC (*asbA/asbB*), AMP-binding (*asbC*), and PP-binding (*asbD*), as well as the Pfam model for DUF6005 (*asbE*, PF19468.2) (59). No *asbF* model was used due to a low bitscore between *mllF* and the expected Pfam model PF01261.27. The multiSMASH workflow (60) was used to scan the downloaded Genbank files for *asbABCDE* homologs and tabulate the results. BGC presence and absence was mapped to the phylogenetic tree with iTOL v6.8 (30). An additional 85 XoxF-containing genomes, listed in Huang et al., 2018, were downloaded from the JGI Genome Portal in paired fasta/gff format and scanned using the same multiSMASH/antiSMASH workflow. Default parameters were used for all software unless otherwise specified. A BGC region met the AsbABCDE rule if it contained matches to existing antiSMASH models for IucA_IucC (*asbA/asbB*), AMP-binding (*asbC*), and PP-binding (*asbD*), as well as the Pfam model for DUF6005 (*asbE*, PF19468.2) (59). No *asbF* model was used due to a low bitscore between *mllF* and the expected Pfam model PF01261.27.

### DNA Manipulation.

Primers used for plasmid and strain construction are listed in *SI Appendix*, Table S2 and Fig. S6. All fragments were amplified with Phusion DNA polymerase (New England Biolabs). Fragments for pMLL were fused using in vivo yeast DNA assembly (61). Competent *Saccharomyces cerevisiae* HZ848 were freshly prepared and transformed with a mixture of purified PCR products and spread on uracil dropout medium (MilliporeSigma). Prototrophic colonies were grown in uracil dropout medium (MilliporeSigma) at 30 °C and 250 rpm and the plasmid was purified using the Yeast Plasmid MiniPrep II Kit (Zymo Research). The plasmid was transformed into TransforMax EPI300TM *Escherichia coli* (Lucigen) and induced according to the manufacturer’s protocol. pMLL was purified using the BAC DNA Miniprep Kit (Zymo Research). pMLL was then electroporated into Δ*mxaF* to generate Δ*mxaF*/pMLL. Fragments for *mll* deletion vector pMS2 were fused using Gibson Assembly Master Mix (New England Biolabs), transformed into competent DH5α *E. coli* (New England Biolabs), and purified using the GeneJET Plasmid Miniprep Kit (Thermo Fischer Scientific). Counterselection was achieved through patching onto Hypho succinate sucrose and Hypho succinate kanamycin plates and selecting colonies with a successful double crossover event (growth on sucrose, no growth on kanamycin). The deletion of the *mll* cluster and absence of suppressor mutations was confirmed by whole genome sequencing (SeqCenter). The *mll* promoter fusion vector pAZ6 was generated through separate double digestions of pTE1877 and the *mll* promoter fragment with AatII and XbaI (New England Biolabs), which were then ligated together using T4 DNA ligase (New England Biolabs) (62). Full plasmid sequencing (Primordium) confirmed the sequence of all constructs (*SI Appendix*, Fig. S6).

### Analytical Scale Identification of Methylolanthanin From Crude Extracts.

50 mL ½ Hypho medium supplemented with 50 mM methanol and 1 μm Nd_2_O_3_ (99.99%, Chempur) was used to culture Δ*mxaF* and Δ*mxaF*/pMLL in 250 mL Erlenmeyer flasks to late-exponential phase (OD_600_ = 0.8). Supernatants were harvested by centrifugation at 4,000×*g* at 4 °C. 25 mL of lyophilized supernatant equivalent were dissolved into 6 mL water and extracted using solid phase on HLB column preparation (MN Chromabond; 60 μm, 500 mg). HLB columns were conditioned with methanol (2 × 6 mL) and then equilibrated with water (2 × 6 mL). The sample was loaded onto the column, the column was washed with water (3 × 6 mL), dried with vacuum, and then eluted with methanol (2 × 6 mL). Methanol was removed in vacuo, the concentrated samples were dissolved in water/acetonitrile (50/50, 1 mL), and the sample was syringe filtered (0.2 μm PTFE).

A 2.5 µL sample was injected into an Agilent QTOF 6530 C coupled to an Agilent HPLC 1260 Infinity II instrument (G7115A 1260 DAD WR, G7116A 1260 MCI, G7167A 1260 multisampler, G7104C 1260 flexible pump). A C18 porous core column (Agilent Poroshell 120 EC-C18, 3.0 × 150 mm, 2.7 μm) was used for chromatography at 30 °C. The mobile phase consisted of solvent A (water + 0.1% formic acid (FA)) and solvent B (acetonitrile + 0.1% FA). The flow rate was set to 0.7 mL/min. After injection, the samples were eluted with the following method: 0 to 2 min 2% B, 2 to 20 min 2 to 98% B, followed by a 6-min washout phase at 98% B and a 3-min re-equilibration phase at 2% B. Data-dependent acquisition (DDA) of MS/MS spectra was performed in both positive and negative modes. ESI, MS, and MS/MS parameters are described in *SI Appendix*.

### Feature Finding in MZmine.

MS/MS spectra were converted to .mzML files using MSConvert (*ProteoWizard*) (63). MS1 feature extraction and MS/MS pairing were performed with MZmine 3 (34). For positive mode data, an intensity threshold of 2.5E2 for MS1 spectra and of 0 for MS/MS spectra was used. MS1 ADAP chromatogram building was performed within a 20 ppm mass window and a minimum highest intensity of 7.5E3 was set. XICs were deconvoluted using the local minimum search algorithm with a chromatographic threshold of 85%, a search minimum in RT range of 0.1 min, and a min ratio of peak top/edge of 1.8. Isotope features were grouped and features from different samples were aligned with 3 ppm mass tolerance and 0.05 min retention time tolerance. MS1 peak lists were joined using an m/z tolerance of 8 ppm and retention time tolerance of 0.4 min; alignment was performed by placing a weight of 75 on m/z and 25 on retention time. Gap filling was performed using an intensity tolerance of 20%, an m/z tolerance of 20 ppm, and a retention tolerance of 0.07 min. Feature areas and the corresponding MS/MS consensus spectra were exported as .csv and .mgf files respectively for postprocessing using R and GNPS.

For negative mode data, an intensity threshold of 6E2 for MS1 spectra and of 0 for MS/MS spectra was used. MS1 ADAP chromatogram building was performed within a 20 ppm mass window and a minimum highest intensity of 1.0E3 was set. XICs were deconvoluted using the local minimum search algorithm with a chromatographic threshold of 87.5%, a search minimum in RT range of 0.1 min, and a min ratio of peak top/edge of 1.8. Isotope features were grouped and features from different samples were aligned with 3 ppm mass tolerance and 0.08 min retention time tolerance. MS1 peak lists were joined using an m/z tolerance of 8 ppm and retention time tolerance of 0.4 min; alignment was performed by placing a weight of 75 on m/z and 25 on retention time. Gap filling was performed using an intensity tolerance of 20%, an m/z tolerance of 20 ppm, and a retention tolerance of 0.08 min. Feature areas and the corresponding MS/MS consensus spectra were exported as .csv and .mgf files respectively for postprocessing using R Stuido (64) and GNPS (33).

### Classical Molecular Networking.

All .mzML files were uploaded to the classical molecular networking workflow in GNPS for spectral networking and spectral library matching (gnps.ucsd.edu). For spectral library matching and spectral networking, the minimum cosine score to define spectral similarity was set to 0.7. The Precursor and Fragment Ion Mass Tolerances were set to 0.02 Da and Minimum Matched Fragment Ions to 4, Minimum Cluster Size to 1 (MS Cluster off). When Analog Search was performed the maximum mass difference was set to 100 Da. The GNPS job for positive analysis can be accessed through the following link: https://gnps.ucsd.edu/ProteoSAFe/status.jsp?task=c9fd99a7d4e147e5a281c23701ac0d2b (molecular family component index 753/cluster index 182991); the negative analysis can be accessed through the following link: https://gnps.ucsd.edu/ProteoSAFe/status.jsp?task=d907c098d05a44b29ed7f670669c2a34 (molecular family component index 131/cluster index 429623).

### Large-Scale Cultivation, Extraction, and Purification of Methylolanthanin.

3 mL cultures of *ΔmxaF*/pMLL were grown in MP medium supplemented with 15 mM succinate and 50 µg/mL kanamycin overnight at 29 °C and 200 rpm in 14 mL vented cap culture tubes (Greiner). Multiple cultures were inoculated into 250 mL of MP medium supplemented with 15 mM succinate and 50 µg/mL kanamycin within a 500 mL Ultra Yield flask (Thompson Instrument) to a starting OD of 0.1 and were grown overnight at 29 °C and 200 rpm. The cells were separated by centrifugation (7 min, 1,800×*g*, r.t.) and washed with ½ Hypho medium (48) supplemented with 125 mM methanol and 50 µg/mL kanamycin. The cells were then resuspended in 5 mL of the same medium and up to five 1 L cultures (½ Hypho, 50 µg/mL kanamycin, 125 mM methanol, 1 μm Nd_2_O_3_) were inoculated at a starting OD of 0.1 in 2.5 L Ultra Yield flasks (Thompson Instrument). The cells were grown at 29 °C and 200 rpm until late stationary phase. The supernatant was obtained as a yellowish liquid after centrifugation (7 min, 15,970×*g*, r.t.) and filtration (0.2 μm, PES).

For large scale solid phase extraction a *puriFlash®* plastic column (60 × 205 mm) was packed with HLB material (MN, 60 μm, 42 g). The column was equilibrated with methanol (2 × 500 mL) and water (2 × 500 mL). Then, up to 2 L of supernatant was loaded onto the column, and the column was washed with water (3 × 500 mL) and fully dried under nitrogen. The column was eluted with methanol (2 × 500 mL) into a 1 L round bottom flask. The solvent was removed in vacuo and the resulting residue was redissolved in water and lyophilized. By this method, an average of 0.07 mg organic matter/mL supernatant was obtained.

HPLC purification was performed on an Agilent 1260 Infinity II preparative HPLC system (G7161A, G7165A, G1364E) equipped with an *Dr. Maisch* ReproSil Gold 120 C18 column (250 × 20 mm, 5 μm). The used solvent system was water/acetonitrile + 0.1% FA. The flow was set to 13.2 mL/min and the method was as follows: isocratic for 2 min at 98/2 water/acetonitrile, then in 40.8 min to 48/52 water/acetonitrile and in 5 min to 2/98 which was held for 10 min; retention time of methylolanthanin: 26 min (*SI Appendix*, Fig. S7). The concentration of a MLL stock solution was determined by qNMR which was then used to obtain the extinction coefficient via UV–Vis (*SI Appendix*, Fig. S8).

### NMR Spectroscopy.

NMR spectra were either recorded on an 800 MHz *Bruker* Avance III HD spectrometer equipped with a triple channel cryogenic probe at 298.15 K or on a 500 MHz *Bruker* BioSpin spectrometer equipped with a broad band observe 5-mm BB-H & CryoProbe^TM^ prodigy at 298.15 K. Spectra were recorded in 3 mm NMR tubes in a 9:1 H_2_O/D_2_O (D_2_O 99.9% + 0.03% TMSP, Deutero) mixture as well as, after lyophilization and redissolving, in D_2_O only. If water suppression was applied the sequence zgesgp was used. All signals were assigned (*SI Appendix*, Fig. S4) using ^1^H, ^1^H-COSY, ^1^H, ^1^H-NOESY, ^1^H, ^1^H-TOCSY and phase-sensitive ^1^H, ^13^C-HSQC and ^1^H, ^13^C-HMBC experiments (*SI Appendix*, Figs. S9–S13). Chemical shifts (*δ*) are reported in parts per million (ppm) using TMSP (0 ppm) as reference.

### Metal-Binding Experiments via Mass Spectrometry.

Metal-binding experiments were performed using an Agilent QTOF 6530 C coupled to an Agilent HPLC 1260 Infinity II instrument (G7115A 1260 DAD WR, G7116A 1260 MCI, G7167A 1260 multisampler, G7104C 1260 flexible pump) via direct injection without a column and instead using a capillary (solvent system 1:1 water/acetonitrile + 0.1 FA, 0.7 mL/min flow) in positive mode. Samples were prepared by mixing methylolanthanin with an excess of LnCl_3_ (Ln = La, Nd, or Lu; purity: 99.99%) in a 1:1 methanol/water mixture.

### Elemental Analysis of Culture Pellets.

50 mL ½ Hypho medium supplemented with 50 mM methanol and 1 μm Nd_2_O_3_ (99.99%, Chempur) was used to culture Δ*mxaF,* Δ*mxaF*/pMLL, and Δ*mxaF*Δ*mll* in 250 mL Erlenmeyer flasks to late-exponential phase (OD600 = 0.8). Cells were pelleted at 4,000×*g* and pellets were washed with water (3 × 10 mL) and resuspended in 1 mL of water and 1 mL of TraceMetal Grade 70% HNO_3_ (Thermo Fisher Scientific). Acidified cell pellets were boiled at 90 °C for 1 h and pelleted at 4,000×*g*. 1 mL of each sample was diluted with 19 mL of water. Samples were analyzed via ICP-MS (Laboratory for Environmental Analysis).

## Supplementary Material

Appendix 01 (PDF)

## Data Availability

The sequencing data from this study have been submitted to the NCBI Gene Expression Omnibus under the Accession No. GSE244060 (20). All MS .raw and centroided .mzML files are publicly available in the mass spectrometry interactive virtual environment (MassIVE) under massive.ucsd.edu with project identifiers MSV000090804 and MSV000083729, available at the following links: ftp://MSV000090804@massive.ucsd.edu and ftp://MSV000090284@massive.ucsd.edu (65, 66). Classical and feature-based molecular networking for positive mode data can be accessed through gnps.ucsd.edu under the following direct links: https://gnps.ucsd.edu/ProteoSAFe/status.jsp?task=c9fd99a7d4e147e5a281c23701ac0d2b (classical MN) and https://gnps.ucsd.edu/ProteoSAFe/status.jsp?task=def58641630e488985bfbe5a15c94e32 (feature-based MN). Classical and feature-based molecular networking for negative mode data can be accessed through gnps.ucsd.edu under the following direct links: https://gnps.ucsd.edu/ProteoSAFe/status.jsp?task=d907c098d05a44b29ed7f670669c2a34 (classical MN) and https://gnps.ucsd.edu/ProteoSAFe/status.jsp?task=0478820eab694254b9eba0bbe4de2be6 (feature-based MN) (67). All R code can be accessed at the following link: https://github.com/allegra-aron/Lanthanophore_2023. All other data are included in the manuscript and/or *SI Appendix*.

## References

[1] E. I. Solomon, U. M. Sundaram, T. E. Machonkin, Multicopper oxidases and oxygenases. Chem. Rev. **96**, 2563–2606 (1996). 11848837 10.1021/cr950046o

[2] C. Andreini, I. Bertini, G. Cavallaro, G. L. Holliday, J. M. Thornton, Metal ions in biological catalysis: From enzyme databases to general principles. J. Biol. Inorg. Chem. **13**, 1205–1218 (2008). 18604568 10.1007/s00775-008-0404-5

[3] K. J. Waldron, J. C. Rutherford, D. Ford, N. J. Robinson, Metalloproteins and metal sensing. Nature **460**, 823–830 (2009). 19675642 10.1038/nature08300

[4] S. M. Kraemer, O. W. Duckworth, J. M. Harrington, W. D. C. Schenkeveld, Metallophores and trace metal biogeochemistry. Aquat. Geochem. **21**, 159–195 (2015).

[5] J. Kramer, Ö. Özkaya, R. Kümmerli, Bacterial siderophores in community and host interactions. Nat. Rev. Microbiol. **18**, 152–163 (2020). 31748738 10.1038/s41579-019-0284-4 PMC7116523

[6] S. Gama , Iron coordination properties of gramibactin as model for the new class of diazeniumdiolate based siderophores. Chemistry **27**, 2724–2733 (2021). 33006390 10.1002/chem.202003842 PMC7898861

[7] L. Qi , Fur in *Magnetospirillum gryphiswaldense* influences magnetosomes formation and directly regulates the genes involved in iron and oxygen metabolism. PLoS ONE **7**, e29572 (2012). 22238623 10.1371/journal.pone.0029572 PMC3251581

[8] K. A. Mettrick, I. L. Lamont, Different roles for anti-sigma factors in siderophore signalling pathways of *Pseudomonas aeruginosa*. Mol. Microbiol. **74**, 1257–1271 (2009). 19889096 10.1111/j.1365-2958.2009.06932.x

[9] B.-E. Ahn , Nur, a nickel-responsive regulator of the Fur family, regulates superoxide dismutases and nickel transport in *Streptomyces coelicolor*. Mol. Microbiol. **59**, 1848–1858 (2006). 16553888 10.1111/j.1365-2958.2006.05065.x

[10] T. Nakagawa , A catalytic role of XoxF1 as La3+-dependent methanol dehydrogenase in *Methylobacterium extorquens* strain AM1. PLoS ONE **7**, e50480 (2012). 23209751 10.1371/journal.pone.0050480 PMC3507691

[11] N. M. Good , Lanthanide-dependent alcohol dehydrogenases require an essential aspartate residue for metal coordination and enzymatic function. J. Biol. Chem. **295**, 8272–8284 (2020). 32366463 10.1074/jbc.RA120.013227 PMC7294098

[12] M. Good Nathan , Pyrroloquinoline quinone ethanol dehydrogenase in *Methylobacterium extorquens* AM1 extends lanthanide-dependent metabolism to multicarbon substrates. J. Bacteriol. **198**, 3109–3118 (2016). 27573017 10.1128/JB.00478-16 PMC5075040

[13] M. Wehrmann, P. Billard, A. Martin-Meriadec, A. Zegeye, J. Klebensberger, Functional role of lanthanides in enzymatic activity and transcriptional regulation of pyrroloquinoline quinone-dependent alcohol dehydrogenases in *Pseudomonas putida* KT2440. mBio **8**, e00570-17 (2017). 28655819 10.1128/mBio.00570-17 PMC5487730

[14] P. Roszczenko-Jasińska , Gene products and processes contributing to lanthanide homeostasis and methanol metabolism in *Methylorubrum extorquens* AM1. Sci. Rep. **10**, 12663 (2020). 32728125 10.1038/s41598-020-69401-4 PMC7391723

[15] A. M. Ochsner , Use of rare-earth elements in the phyllosphere colonizer *Methylobacterium extorquens* PA1. Mol. Microbiol. **111**, 1152–1166 (2019). 30653750 10.1111/mmi.14208 PMC6850437

[16] A. E. Taunton, S. A. Welch, J. F. Banfield, Microbial controls on phosphate and lanthanide distributions during granite weathering and soil formation. Chem. Geol. **169**, 371–382 (2000).

[17] A. D. Kotelnikova, O. B. Rogova, V. V. Stolbova, Lanthanides in the soil: Routes of entry, content, effect on plants, and genotoxicity (a review). Eurasian Soil Sci. **54**, 117–134 (2021).

[18] J. H. L. Voncken, “The ore minerals and major ore deposits of the rare earths” in The Rare Earth Elements: An Introduction, J. H. Voncken, Eds. (Springer International Publishing, 2016), pp. 15–52. 10.1007/978-3-319-26809-5_2.

[19] N. Vu Huong , Lanthanide-dependent regulation of methanol oxidation systems in *Methylobacterium extorquens* AM1 and their contribution to methanol growth. J. Bacteriol. **198**, 1250–1259 (2016). 26833413 10.1128/JB.00937-15 PMC4859578

[20] A. Zytnick, GSE244060: Discovery and characterization of the first known biological lanthanide chelator. NCBI GEO. https://www.ncbi.nlm.nih.gov/geo/query/acc.cgi?acc=GSE244060. Deposited 26 September 2023.

[21] N. M. Good, R. S. Moore, C. J. Suriano, N. C. Martinez-Gomez, Contrasting in vitro and in vivo methanol oxidation activities of lanthanide-dependent alcohol dehydrogenases XoxF1 and ExaF from *Methylobacterium extorquens* AM1. Sci. Rep. **9**, 4248 (2019). 30862918 10.1038/s41598-019-41043-1 PMC6414531

[22] W. Gu, J. D. Semrau, Copper and cerium-regulated gene expression in *Methylosinus trichosporium* OB3b. Appl. Microbiol. Biotechnol. **101**, 8499–8516 (2017). 29032471 10.1007/s00253-017-8572-2

[23] S. Masuda , Lanthanide-dependent regulation of methylotrophy in *Methylobacterium aquaticum* strain 22A. mSphere **3**, e00462-17 (2018). 10.1128/mSphere.00462-17 PMC5784242 29404411

[24] F. Chu, M. E. Lidstrom, XoxF acts as the predominant methanol dehydrogenase in the type I methanotroph *Methylomicrobium buryatense*. J. Bacteriol. **198**, 1317–1325 (2016). 26858104 10.1128/JB.00959-15 PMC4859581

[25] O. Baars, F. M. M. Morel, X. Zhang, The purple non-sulfur bacterium *Rhodopseudomonas palustris* produces novel petrobactin-related siderophores under aerobic and anaerobic conditions. Environ. Microbiol. **20**, 1667–1676 (2018), 10.1111/1462-2920.14078. 29473283

[26] J. Y. Lee , Biosynthetic analysis of the petrobactin siderophore pathway from *Bacillus anthracis*. J. Bacteriol. **189**, 1698–1710 (2007). 17189355 10.1128/JB.01526-06 PMC1855748

[27] M. A. Llamas, F. Imperi, P. Visca, I. L. Lamont, Cell-surface signaling in *Pseudomonas*: Stress responses, iron transport, and pathogenicity. FEMS Microbiol. Rev. **38**, 569–597 (2014). 24923658 10.1111/1574-6976.12078

[28] B. Sachman-Ruiz , IurV, encoded by ORF VCA0231, is involved in the regulation of iron uptake genes in *Vibrio cholerae*. Genes **11**, 1184 (2020). 33053678 10.3390/genes11101184 PMC7600106

[29] F. Asnicar , Precise phylogenetic analysis of microbial isolates and genomes from metagenomes using PhyloPhlAn 3.0. Nat. Commun. **11**, 2500 (2020). 32427907 10.1038/s41467-020-16366-7 PMC7237447

[30] I. Letunic, P. Bork, Interactive tree of life (iTOL) v5: An online tool for phylogenetic tree display and annotation. Nucleic Acids Res. **49**, W293–W296 (2021). 33885785 10.1093/nar/gkab301 PMC8265157

[31] K. Blin , antiSMASH 7.0: New and improved predictions for detection, regulation, chemical structures and visualisation. Nucleic Acids Res. **51**, W46–W50 (2023), 10.1093/nar/gkad344. 37140036 PMC10320115

[32] J. Huang, Z. Yu, L. Chistoserdova, Lanthanide-dependent methanol dehydrogenases of Xox4 and Xox5 clades are differentially distributed among methylotropic bacteria and they reveal different biochemical properties. Front. Microbiol. **9**, 1366 (2018). 29997591 10.3389/fmicb.2018.01366 PMC6028718

[33] M. Wang , Sharing and community curation of mass spectrometry data with Global Natural Products Social Molecular Networking. Nat. Biotechnol. **34**, 828–837 (2016). 27504778 10.1038/nbt.3597 PMC5321674

[34] R. Schmid , Integrative analysis of multimodal mass spectrometry data in MZmine 3. Nat. Biotechnol. **41**, 447–449 (2023). 36859716 10.1038/s41587-023-01690-2 PMC10496610

[35] N. M. Good , Hyperaccumulation of gadolinium by *Methylorubrum extorquens* AM1 reveals impacts of lanthanides on cellular processes beyond methylotrophy. Front. Microbiol. **13**, 820327 (2022). 35369483 10.3389/fmicb.2022.820327 PMC8969499

[36] M. Y. Voutsinos, J. A. West-Roberts, R. Sachdeva, J. W. Moreau, J. F. Banfield, Do lanthanide-dependent microbial metabolisms drive the release of REEs from weathered granites? bioRxiv [Preprint] (2022). 10.1101/2022.03.08.483559 (Accessed 5 September 2023).

[37] M. Wehrmann, C. Berthelot, P. Billard, J. Klenbensberger, Rare earth element (REE)-dependent growth of *Pseudomonas putida* KT2440 relies on the ABC-transporter PedA1A2BC and is influenced by iron availability. Front. Microbiol. **10**, 2494 (2019). 31736923 10.3389/fmicb.2019.02494 PMC6839425

[38] K. Mehdiratta , Kupyaphores are zinc homeostatic metallophores required for colonization of *Mycobacterium tuberculosis*. Proc. Natl. Acad. Sci. U.S.A. **119**, e2110293119 (2022). 35193957 10.1073/pnas.2110293119 PMC8872721

[39] E. Butaitė, M. Baumgartner, S. Wyder, R. Kümmerli, Siderophore cheating and cheating resistance shape competition for iron in soil and freshwater *Pseudomonas* communities. Nat. Commun. **8**, 414 (2017). 28871205 10.1038/s41467-017-00509-4 PMC5583256

[40] H. Lumpe , The earlier the better: Structural analysis and separation of lanthanides with pyrroloquinoline quinone. Chem. Eur. J. **26**, 10133–10139 (2020). 32497263 10.1002/chem.202002653 PMC7496819

[41] H. Lumpe, L. J. Daumann, Studies of redox cofactor pyrroloquinoline quinone and its interaction with lanthanides(III) and calcium(II). Inorg. Chem. **58**, 8432–8441 (2019). 31184864 10.1021/acs.inorgchem.9b00568

[42] H. Toyama, M. E. Lidstrom, pqqA is not required for biosynthesis of pyrroloquinoline quinone in *Methylobacterium extorquens* AM1. Microbiology **144**, 183–191 (1998). 9467911 10.1099/00221287-144-1-183

[43] X. Ge , Multiple pqqA genes respond differently to environment and one contributes dominantly to pyrroloquinoline quinone synthesis. J. Basic Microbiol. **55**, 312–323 (2015). 23828377 10.1002/jobm.201300037

[44] N. M. Good , Scalable and consolidated microbial platform for rare earth element leaching and recovery from waste sources. Environ. Sci. Technol. **58**, 570–579 (2023). 38150661 10.1021/acs.est.3c06775 PMC10785750

[45] B. F. Pfleger , Structural and functional analysis of AsbF: Origin of the stealth 3,4-dihydroxybenzoic acid subunit for petrobactin biosynthesis. Proc. Natl. Acad. Sci. U.S.A. **105**, 17133–17138 (2008). 18955706 10.1073/pnas.0808118105 PMC2579390

[46] D. N. Nunn, M. E. Lidstrom, Isolation and complementation analysis of 10 methanol oxidation mutant classes and identification of the methanol dehydrogenase structural gene of *Methylobacterium* sp. strain AM1. J. Bacteriol. **166**, 581–590 (1986). 3009411 10.1128/jb.166.2.581-590.1986 PMC214644

[47] C. J. Marx, B. N. O’Brien, J. Breezee, M. E. Lidstrom, Novel methylotrophy genes of *Methylobacterium extorquens* AM1 identified by using transposon mutagenesis including a putative dihydromethanopterin reductase. J. Bacteriol. **185**, 669–673 (2003). 12511515 10.1128/JB.185.2.669-673.2003 PMC145341

[48] N. F. Delaney , Development of an optimized medium, strain and high-throughput culturing methods for *Methylobacterium extorquens*. PLoS ONE **8**, e62957 (2013). 23646164 10.1371/journal.pone.0062957 PMC3639900

[49] N. M. Good, N. C. Martinez-Gomez, D. A. C. Beck, M. E. Lidstrom, Ethylmalonyl coenzyme A mutase operates as a metabolic control point in *Methylobacterium extorquens* AM1. J. Bacteriol. **197**, 727–735 (2015). 25448820 10.1128/JB.02478-14 PMC4334189

[50] A. P. Arkin , KBase: The United States Department of Energy Systems biology knowledgebase. Nat. Biotechnol. **36**, 566–569 (2018). 29979655 10.1038/nbt.4163 PMC6870991

[51] S. R. Eddy, Accelerated profile HMM searches. PLoS Comput. Biol. **7**, e1002195 (2011). 22039361 10.1371/journal.pcbi.1002195 PMC3197634

[52] S. C. Potter , HMMER web server: 2018 update. Nucleic Acids Res. **46**, W200–W204 (2018). 29905871 10.1093/nar/gky448 PMC6030962

[53] M. Johnson , NCBI BLAST: A better web interface. Nucleic Acids Res. **36**, W5–W9 (2008). 18440982 10.1093/nar/gkn201 PMC2447716

[54] D. H. Haft , RefSeq: An update on prokaryotic genome annotation and curation. Nucleic Acids Res. **46**, D851–D860 (2018). 29112715 10.1093/nar/gkx1068 PMC5753331

[55] K. Blin, NCBI-Genome-Download (Zenodo, 2023). 10.5281/ZENODO.8192432.

[56] K. Katoh, D. M. Standley, MAFFT multiple sequence alignment software version 7: Improvements in performance and usability. Mol. Biol. Evol. **30**, 772–780 (2013). 23329690 10.1093/molbev/mst010 PMC3603318

[57] A. Stamatakis, RAxML version 8: A tool for phylogenetic analysis and post-analysis of large phylogenies. Bioinformatics **30**, 1312–1313 (2014). 24451623 10.1093/bioinformatics/btu033 PMC3998144

[58] B. Buchfink, C. Xie, D. H. Huson, Fast and sensitive protein alignment using DIAMOND. Nat. Methods **12**, 59–60 (2015). 25402007 10.1038/nmeth.3176

[59] S. El-Gebali , The Pfam protein families database in 2019. Nucleic Acids Res. **47**, D427–D432 (2019). 30357350 10.1093/nar/gky995 PMC6324024

[60] Z. L. Reitz, multiSMASH (Zenodo, 2023). 10.5281/zenodo.8276144.

[61] Z. Shao, H. Zhao, H. Zhao, DNA assembler, an in vivo genetic method for rapid construction of biochemical pathways. Nucleic Acids Res. **37**, e16 (2009). 19074487 10.1093/nar/gkn991 PMC2632897

[62] M. Carrillo , Design and control of extrachromosomal elements in *Methylorubrum extorquens* AM1. ACS Synth. Biol. **8**, 2451–2456 (2019). 31584803 10.1021/acssynbio.9b00220 PMC6862569

[63] M. C. Chambers , A cross-platform toolkit for mass spectrometry and proteomics. Nat. Biotechnol. **30**, 918–920 (2012). 23051804 10.1038/nbt.2377 PMC3471674

[64] RStudio Team, RStudio: Integrated Development for R (RStudio Team, 2020).

[65] S. Gutenthaler, MSV000090804: GNPS non-targeted MS data of *M. extorquens* AM1 in search of lanthanophores. MassIVE. 10.25345/C50K26G78. Deposited 29 November 2022.

[66] A. T. Aron, MSV000090284: GNPS - 09082022_Methylorubrum_extorquens_AM1__HLB50,80,100. MassIVE. 10.25345/C5TM7253B. Deposited 8 September 2022.

[67] A. T. Aron, Lanthanophore_2023: Release_1. Zenodo. 10.5281/zenodo.12735162. Deposited 12 July 2024.

