# Supplementary material for: Identification and characterization of a small-molecule metallophore involved in lanthanide metabolism
Source: Proc Natl Acad Sci U S A. 2024 Jul 30;121(32):e2322096121. doi: 10.1073/pnas.2322096121 (PMC11317620; doi:10.1073/pnas.2322096121)
Supplement: Supplementary file 1 — Appendix 01 (PDF) [file pnas.2322096121.sapp.pdf]

PNAS

Supporting Information for

Discovery and characterization of the first small molecule metallophore involved in lanthanide metabolism

Alexa M. Zytnick, Sophie M. Gutenthaler-Tietze, Allegra T. Aron, Zachary L. Reitz, Manh Tri Phi, Nathan M. Good, Daniel Petras, Lena J. Daumann, Norma Cecilia Martinez-Gomez.

*Norma Cecilia Martinez-Gomez, Lena J. Daumann
Email: cecimartinez@berkeley.edu, lena.daumann@hhu.de

This PDF file includes:

Supporting text
Figures S1 to S13
Tables S1 to S2

Supporting Information Text

Methods

Identification of methylolanthanin at analytical scale from crude extracts. ESI parameters were set to the following: (positive mode) Gas Temp: 250 °C, Drying Gas: 11 L/min, Nebuliser 45 psi, Sheath Gas Temp 350 °C, Sheath Gas Flow 12 L/min; VCap 3500 V, Fragmentor 100 V, Skimmer 65 V, Oct 1 RF Vpp 750 V; mass range: 100-1700; rate 5 spectra/s; time 200 ms/spectrum; transients/spectrum 2641; Reference masses used: 121.0509 and 922.0098; (negative mode) Gas Temp: 275 °C, Drying Gas: 11 L/min, Nebuliser 35 psi, Sheath Gas Temp 350 °C, Sheath Gas Flow 12 L/min; VCap 3000 V, Fragmentor 100 V, Skimmer 65 V, Oct 1 RF Vpp 750 V; rate 5 spectra/s; time 200 ms/spectrum; transients/spectrum 2677; Reference masses used: 112.9855 and 1033.9881.

MS/MS settings for iterative aMS/MS were the following and were measured from 2 to 22 min: Spectral Parameters: (MS 100-1700 m/z; rate 4 spectra/s; time 250; transients/spectrum 3330; MS/MS 50-1000 m/z, rate 4, time 250, transients/spectrum 3211; isolation width ~1.2 m/z); cycle time 0.85 seconds

Collision Energy - fixed: 10, 20 and 40 V; Precursor Selection I : 2 per cycle; Threshold 2000 counts (relative Threshold 0.01%); Active Exclusion after 2 Spectra, released after 0.1 min; (the reference molecules were excluded); Settings for iterative MS/MS: mass tolerance +/- 20 ppm; retention time exclusion tolerance +/-0.2 min; Precursor Selection II: Isotope Model: unbiased; Charge State selection: 1, 2, Unk; Abundance Dependent Accumulation - Scan Speed varied based on precursor abundance.

Solid phase extraction for Q Exactive HF. 50 mL of lyophilized supernatant were reconstituted in 6 mL of 3% methanol/LCMS grade water and was extracted onto HLB cartridges (MN Chromabond; 60 µM, 500 mg). HLB cartridges were pre-activated with methanol (2x3 mL), then were washed with water + 3% methanol (2x3 mL). Samples were loaded dropwise onto SPE cartridges before cartridges were washed with water + 3% methanol (2x3 mL). Then samples were eluted stepwise into 2 mL of 50% methanol, 2 mL of 80% methanol, 2 mL of 100% methanol. Samples were weighed and reconstituted with 100 µL 80% methanol/20% water.

UHPLC-MS/MS on Q Exactive HF. For data-dependent UHPLC-MS/MS analysis, 5 µL of sample were injected per run. A C18 EVO porous core column (Kinetex C18, 50 × 2 mm, particle size of 1.8 µm, pore size of 100 Å, Phenomenex) was used for reversed-phase chromatography. A Vanquish high-pressure binary gradient system couple to a Q Exactive HF mass spectrometer and method according to Stincone *et al.* was used. The mobile phase consisted of solvent A (water+ 0.1% formic acid (FA)) and solvent B (acetonitrile (ACN) + 0.1% FA), and the flow rate was 0.5 ml min^{-1}. After injection, the samples were eluted with the linear gradient: 0–8 min 5–50% B, followed an increase from 50-99 % B from 8-12 min, and by a 3-min washout phase at 99% B and a 3-min re-equilibration phase at 5% B. Data-dependent acquisition (DDA) of MS/MS spectra was performed in positive mode. ESI parameters were set to a sheath gas flow of 50AU, auxiliary gas flow of 12 AU, sweep gas flow of I AU and auxiliary gas temperature of 400 °C, while the spray voltage was set to 3.5 kV, the inlet capillary to 250 °C. The MS scan range was set to 150–1,500 m/z with a resolution at m/z 200 (Rm/z 200) of 120,000. The maximum ion injection time was set to 100 ms with an automated gain control (AGC) target of 1.0×10^6. Up to five MS/MS spectra per MS1 survey scan were recorded in DDA mode with Rm/z 200 of 15,000 with one micro-scan. The maximum ion injection time for MS/MS scans was set to 50 ms with an AGC target of 5×10^5 ions. The MS/MS precursor isolation window was set to m/z 1. The normalized collision energy was set to a stepwise increase from 25 to 35 to 45% with z = 1 as default charge state. MS/MS scans were triggered at the apex of chromatographic peaks within 2 to 15 s from their first occurrence. Dynamic precursor exclusion was set to 5 s. Ions with unassigned charge states were excluded from MS/MS acquisition as well as isotope peaks.

Feature based molecular networking. The .csv and .mgf file outputs from Feature finding in MZmine were directly uploaded to the feature based molecular networking workflow in GNPS for spectral networking and spectral library matching (gnps.ucsd.edu). For spectral library matching and spectral networking, the minimum cosine score to define spectral similarity was set to 0.7. The Precursor and Fragment Ion Mass Tolerances were set to 0.02 Da and Minimum Matched Fragment Ions to 4, Minimum Cluster Size to 1 (MS Cluster off). When Analog Search was performed the maximum mass difference was set to 100 Da. The GNPS job for positive analysis can be accessed through the following link: https://gnps.ucsd.edu/ProteoSAFe/status.jsp?task=def58641630e488985bfbe5a15c94e32; the negative analysis can be accessed through the following link: https://gnps.ucsd.edu/ProteoSAFe/status.jsp?task=0478820eab694254b9eba0bbe4de2be6.

LC-MS/MS data cleanup and statistics. The .csv feature tables from Feature finding in MZmine 3 were blank subtracted, imputed, normalized, and scaled using R code available at https://github.com/allegra-aron/Lanthanophore_2023/. A media blank was used for blank subtraction, specifically features with mean intensity of 30% abundance in the media as compared to samples were removed. Imputation was utilized to remove zero values by replacing them with the limit of detection for the experiment. Normalization was performed by scaling the intensities of all features in a sample by the total ion current (or sum) of the sample (total ion current or TIC normalization). Volcano plots and Kruskal-Wallis analysis followed by pairwise Wilcoxon tests and Benjamini-Hochberg (BH) correction were performed using R code provided in the same location (https://github.com/allegra-aron/Lanthanophore_2023/). The blank subtracted, imputed, TIC normalized table was utilized for volcano plots and Kruskal-Wallis analysis. Principal component analysis was performed using the scaled data.

Concentration determination *via* qNMR and determination of the extinction coefficient. ^{1}H qNMR was measured in 3 mm NMR tubes (*Deutero*, D800-3-7) on a *Bruker* Avance III HD spectrometer equipped with a triple channel cryogenic probe operating at the Larmor frequency of 800 MHz (18.8 T) at 298.15 K. The following acquisition settings were used: spectral width of 12820.5 Hz, 48 scans, acquisition time of 1.278 s, 60 s relaxation delay and 16 k real points. For water suppression the pulse sequence zgesgp was used. A stock solution (10 mg/mL; MW: 130.11 g/mol) in D_2O (+ 0.03% TMSP, *Deutero*) of the quantitative reference material calcium formate (*Merck*; certified reference material, 99.58%) was gravimetrically prepared (*Mettler Toledo* UMT2 precision scale, Δ=0.1µg) and diluted 1:10 with D_2O + 0.03% TMS before it was mixed 1:10 with an aqueous solution with an unknown concentration of methylolanthanin. The equation (1) was used to calculate the concentration (c_x) of methylolanthanin. With I standing for integral, N for number of atomic nuclei and P_{Std} for purity (%). The qNMR spectrum and the used integral values are shown in Fig. S8.

$$(1)\quad c_x = \frac{I_x}{I_{Std}} \times \frac{N_{Std}}{N_x} \times c_{Std} \times P_{Std}$$

With this the concentration of the aqueous solution of methylolanthanin was determined and was usedto prepare a series of samples in water with known concentration to determine the extinction coefficient (ε) using the *Beer-Lambert* law (Fig. S8). UV-vis spectra were recorded on an *Agilent* Cary 60 spectrometer at 25 °C in *Brand* micro cuvettes (10 mm pathlength; 70-500 µL) with a scan rate of 600 nm/min in 1 nm steps. All spectra were measured using the automatic baseline correction after measuring the used solvent. In order to determine the extinction coefficient, the absorbance at 251 nm was plotted against the concentration which gave an extinction coefficient of 24.3 +/– 0.3 $mM^{-1}cm^{-1}$.

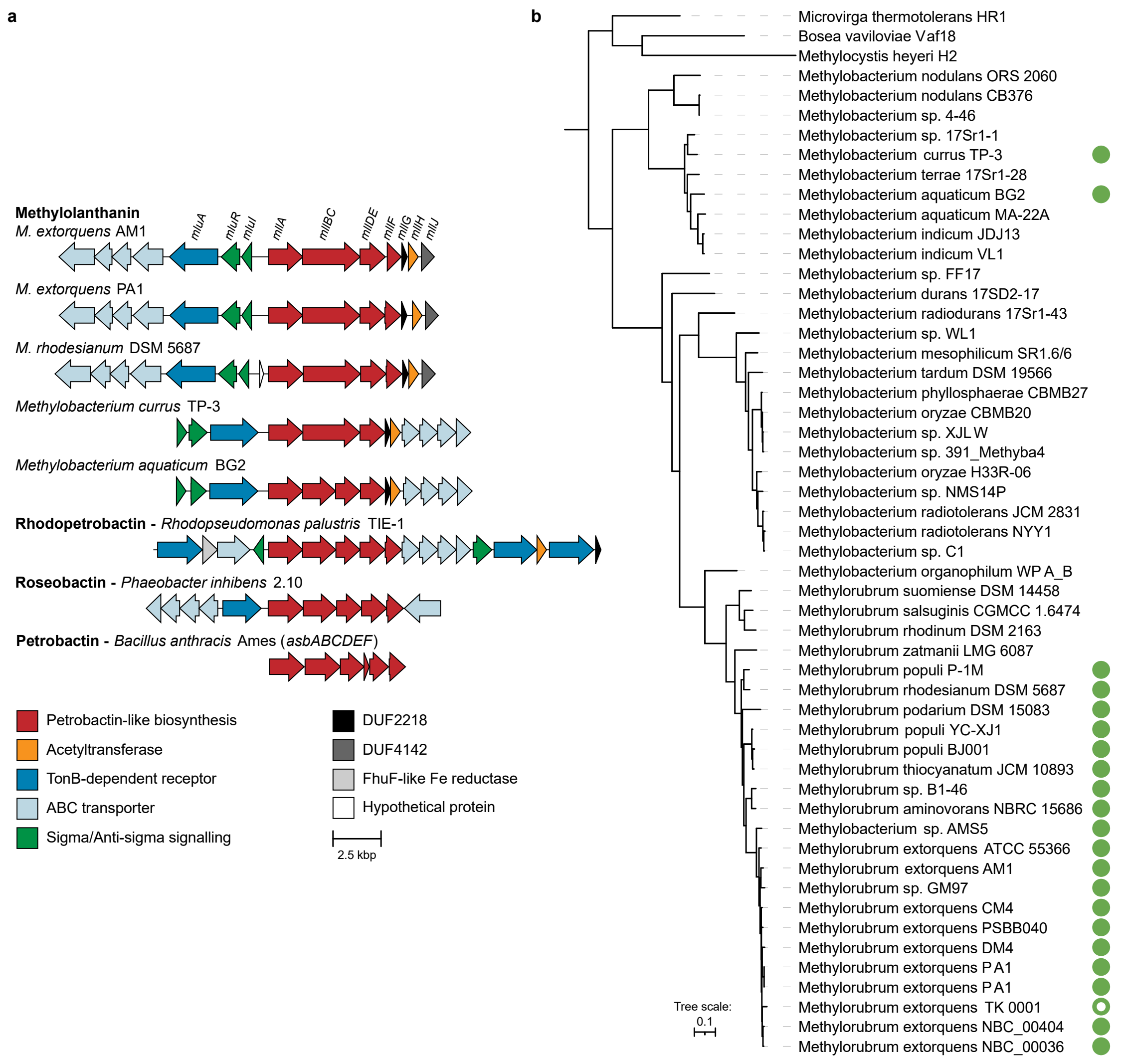

Figure S1. Comparative genomics of *mll* and prevalence in *Methylobacteriaceae*. a, Comparison of *mll* from *Methylorubrum exotorquens* AM1 against homologous clusters, including BGCs encoding the characterized siderophores rhodopetrobactin, roseobactin, and petrobactin. Genes are drawn to scale. **b,** A maximum-likelihood phylogenetic species tree of *Methylorubrum* and *Methylobacterium* strains, annotated by the presence of a BGC homologous to *mll* (filled green circle). The *asbC* homolog in *M. extorquens* TK 0001 (open circle) did not match the AMP-binding domain (PF00501) and may be non-functional. Assembly accession numbers are provided in Supplementary Table 1.

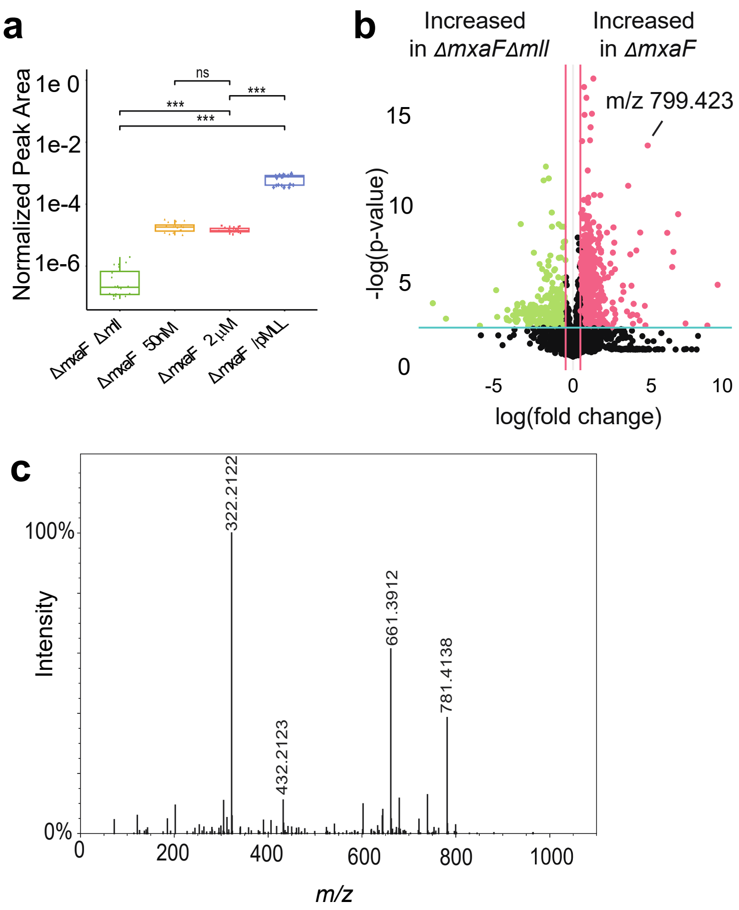

Figure S2. Statistical analysis and MS/MS of methylolanthanin. **a**, Boxplots of the normalized peak area of feature *m/z* 799.4232 in positive mode reveal significant differences between Δ*mxaF* and Δ*mxaF*Δ*mll* and between Δ*mxaF*/pMLL but differences between Δ*mxaF* 2 μM Nd and Δ*mxaF* 50 nM were not significant. Statistics were calculated with n=5 per group. Kruskal-Wallis, followed by pairwise Wilcoxon tests and Benjamini-Hochberg (BH) correction, was used (***p < 0.001). Upper and lower whiskers extend to closest value to +/- 1.5 * IQR. **b**, *m/z* 799.4232 is one of the most significantly increased features in Δ*mxaF* versus Δ*mxaF*Δ*mll* supernatants when analyzed using ESI-UPLC-MS/MS in negative ionization mode. Volcano plot analysis was performed with n=20 per group. Fold changes 0.5 and a p-value < 0.01 are highlighted. **c**, The MS/MS spectrum in positive mode of the 1+ methylolanthanin.

a

1H NMR chemical shifts (ppm) for Methylolanthanin				
C/H	δH (D_2O)	Mult. (J in Hz)	δC (D_2O)	HMBC
1	-	-	162.6	
2	6.94	8.6 (d)	118.3	C1, C2, C4
3	7.67	8.6 (d)	132.3	C1, C3, C5
4	-	-	128.7	
5	-	-	173.4	
6	-	-	-	-
7	3.36, 3.38	m	48.3, 42.2	C5, C8, C9
8/9	1.47, 1.57; 1.42, 1.58	m, m	27.1, 28.6	
10	3.29, 3.33	m	51.8	C11
11	3.25, 3.30	m	48.6	
12	-	-	176.5	
13	2.07, 2.07	s, s	23.3	C12
14	1.48, 1.56	m	27.2, 27.3	
15	1.39, 1.43	m	28.7, 28.6	
16	3.08, 3.09	m	41.7	C18
17	-	-	-	
18	-	-	175.1	
19	HA 2.66<br>HB 2.56	14.4 (d, 4 AB systems overlapping)	47.2	C18, C19, C20, C21
20	-	-	77.9	
21	-	-	182.0	
C/H	δH (H_2O/D_2O)	Mult. (J in Hz)	δC (H_2O/D_2O)	HMBC
6	8.31, 8.28	6.3 (t), 6.2 (t)	-	
17	7.81, 7.80	5.9 (t), 6.0 (t)	-	
19	HA 2.68<br>HB 2.57	14.7 (d, 4 AB systems overlapping)	47.2	C18, C19, C20, C21

b

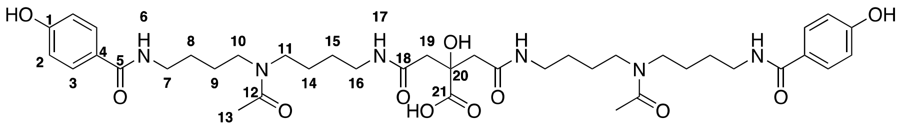

Figure S3. NMR assignments of methylolanthanin. a, ^{1}H NMR chemical shifts (ppm) for methylolanthanin. **b**, Structure of methylolanthanin with numbering for NMR assignments.

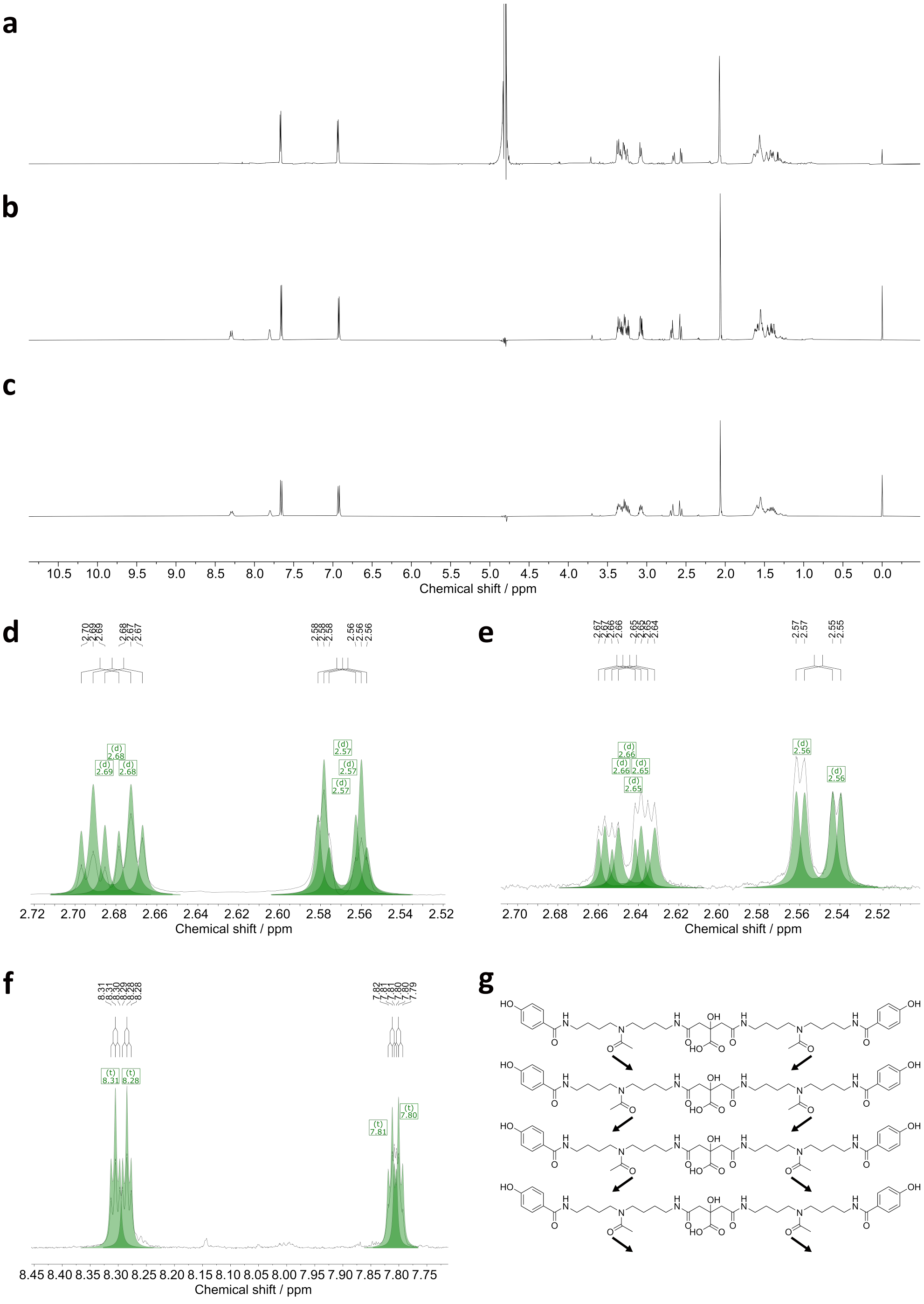

Figure S4. **^{1}H NMR spectra of methylolanthanin**. **a,** in D_2O (800 MHz), **b,** in H_2O/D_2O (9:1) (800 MHz) and **c,** in H_2O/D_2O (9:1) (500 MHz). **d,** Deconvoluted signals observed for the four overlapping AB spin systems of the diastereotopic methylene signals of the central citric acid moiety of methylolanthanin in D_2O/H_2O (800 MHz) and **e,** in D_2O (800 MHz). **f,** deconvoluted signals observed for the secondary amines protons of methylolanthanin in D_2O/H_2O (800 MHz) caused by the proximity to differently orientated acyl groups. **g,** Molecular structures of the four possible conformers of methylolanthanin causing the signal splitting.

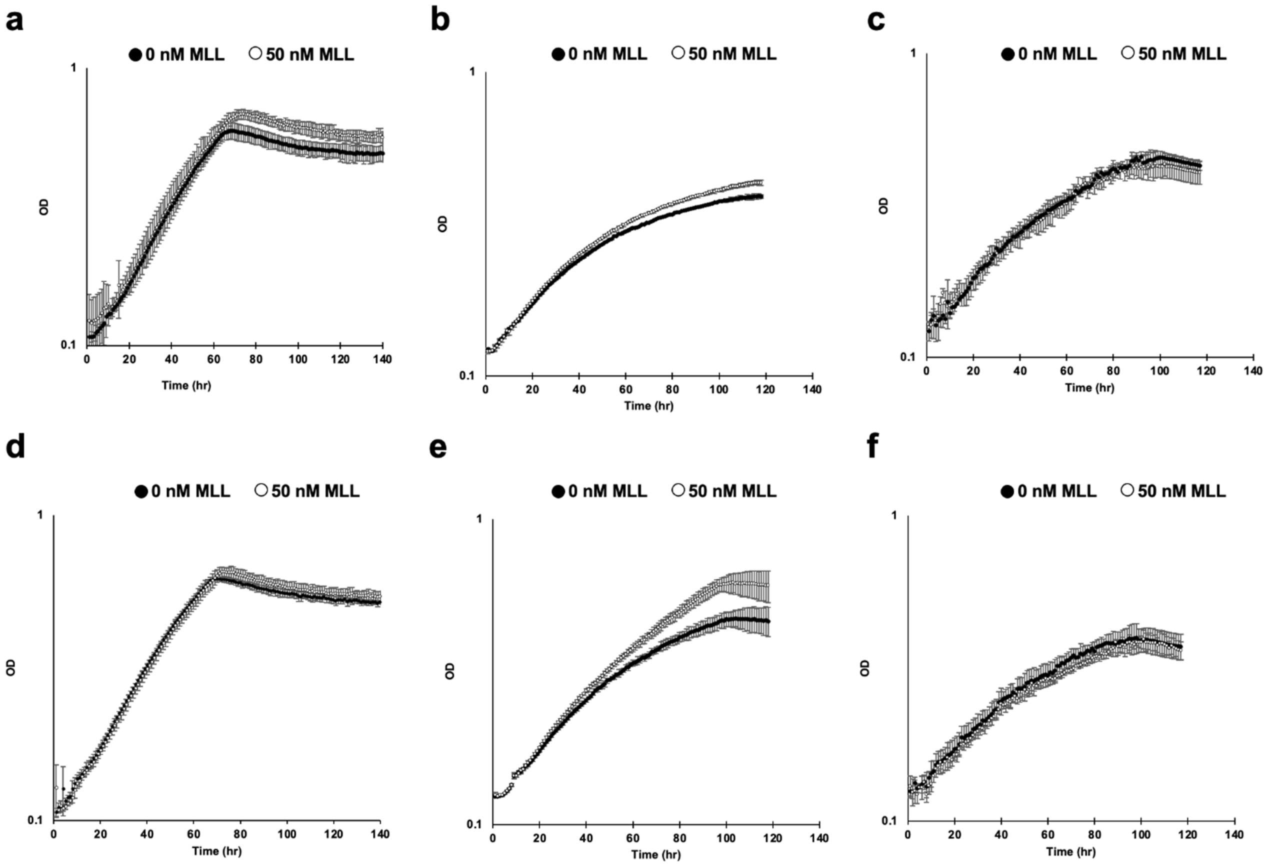

Figure S5. Exogenous methylolanthanin affects growth of Δ*mxaF* and Δ*mxaF*Δ*mlI*. **a**, Addition of methylolanthanin to Δ*mxaF* cultures (empty circles) compared to cultures grown without methylolanthanin (filled circles) when grown with 2 μM $NdCl_3$. **b**, Addition of methylolanthanin to Δ*mxaF* cultures (empty circles) compared to cultures grown without methylolanthanin (filled circles) when grown with 50 nM $NdCl_3$. **c**, Addition of methylolanthanin to Δ*mxaF* cultures (empty circles) compared to cultures grown without methylolanthanin (filled circles) when grown with 1 μM Nd_2O_3. **d**, Addition of methylolanthanin to Δ*mxaFmlI* cultures (empty circles) compared to cultures grown without methylolanthanin (filled circles) when grown with 2 μM $NdCl_3$. **e**, Addition of methylolanthanin to Δ*mxaFmlI* cultures (empty circles) compared to cultures grown without methylolanthanin (filled circles) when grown with 50 nM $NdCl_3$. **f**, Addition of methylolanthanin to Δ*mxaFmlI* cultures (empty circles) compared to cultures grown without methylolanthanin (filled circles) when grown with 1 μM Nd_2O_3. Individual data points represent the mean of three replicates and error bars represent standard deviation.

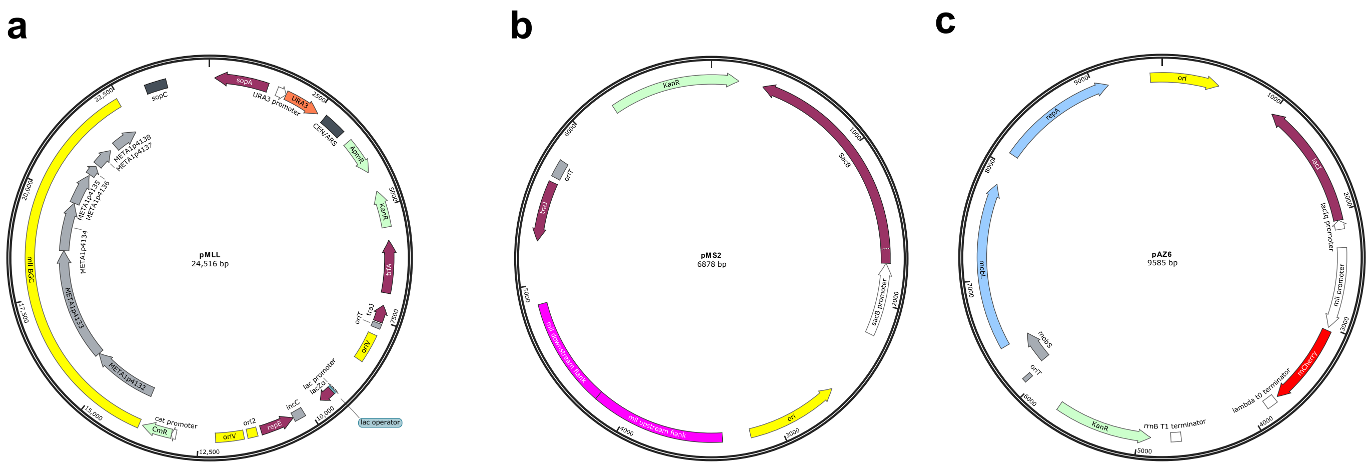

Figure S6. Plasmids used in study. a, Plasmid map of pMLL used to generate the *mll* overexpression mutant. pMLL is a high copy number plasmid encoding the full *mll* BGC under the control of the lac promoter. **b**, Plasmid map of pMS2 used to generate the *mll* deletion mutant. pMS2 is an allelic exchange vector encoding the *sacB* gene as well as upstream and downstream flanks of the *mll* BGC to facilitate deletion of the full *mll* cluster. **c,** Plasmid map of pAZ6 used to generate *mll* promoter fusion strains. pAZ6 contains the region upstream of *mll* fused to mCherry for fluorescence measurement of *mll* promoter activity.

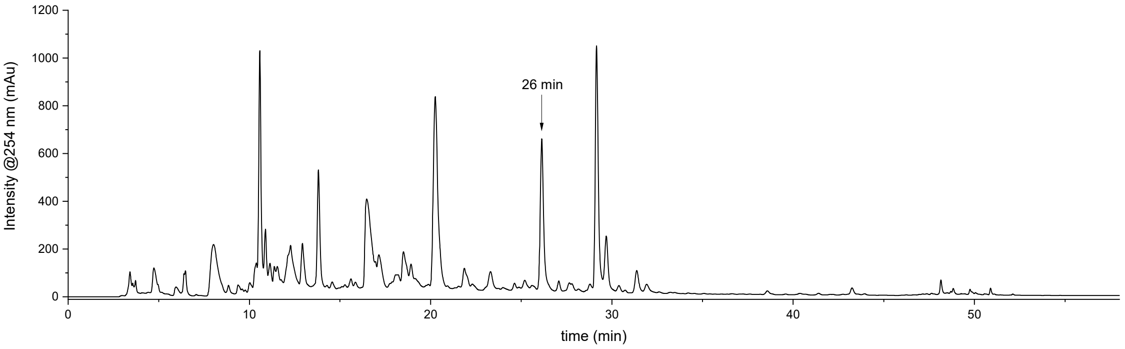

Figure S7. Preparative HPLC chromatogram of methylolanthanin. Extraction from culture supernatants ran on reversed-phase column shows elution of methylolanthanin at 26 minutes.

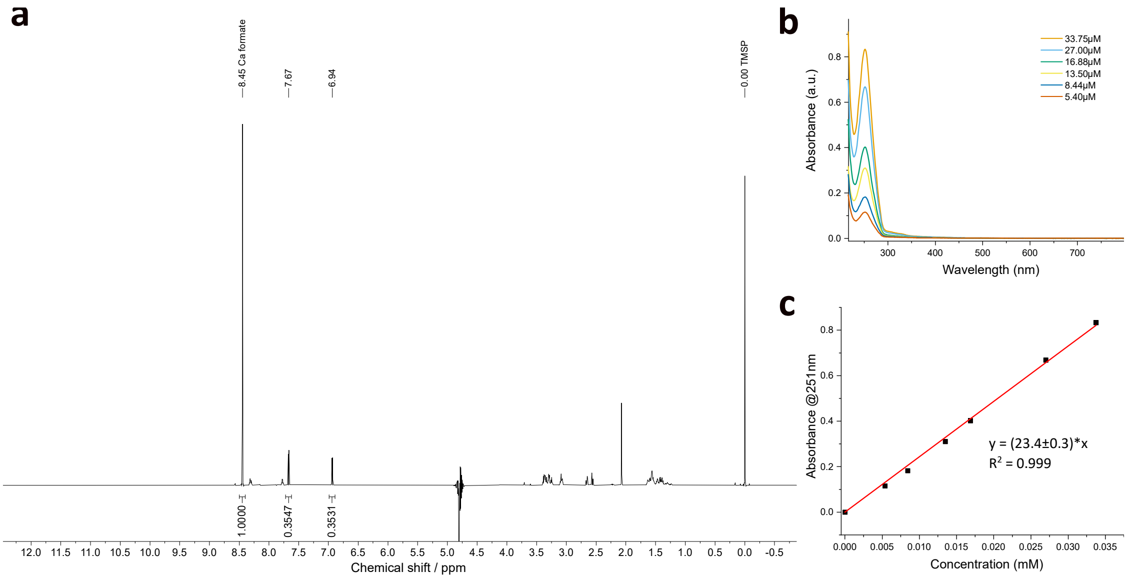

Figure S8. qNMR and extinction coefficient determination of methylolanthanin. a, qNMR of methylolanthanin in H_2O/D_2O (9:1) (800MHz). **b**, UV-vis spectra of methylolanthanin at different concentrations in water. **c**, Concentration of methylolanthanin plotted against the absorbance at 251 nm for determination of the extinction coefficient from regression line.

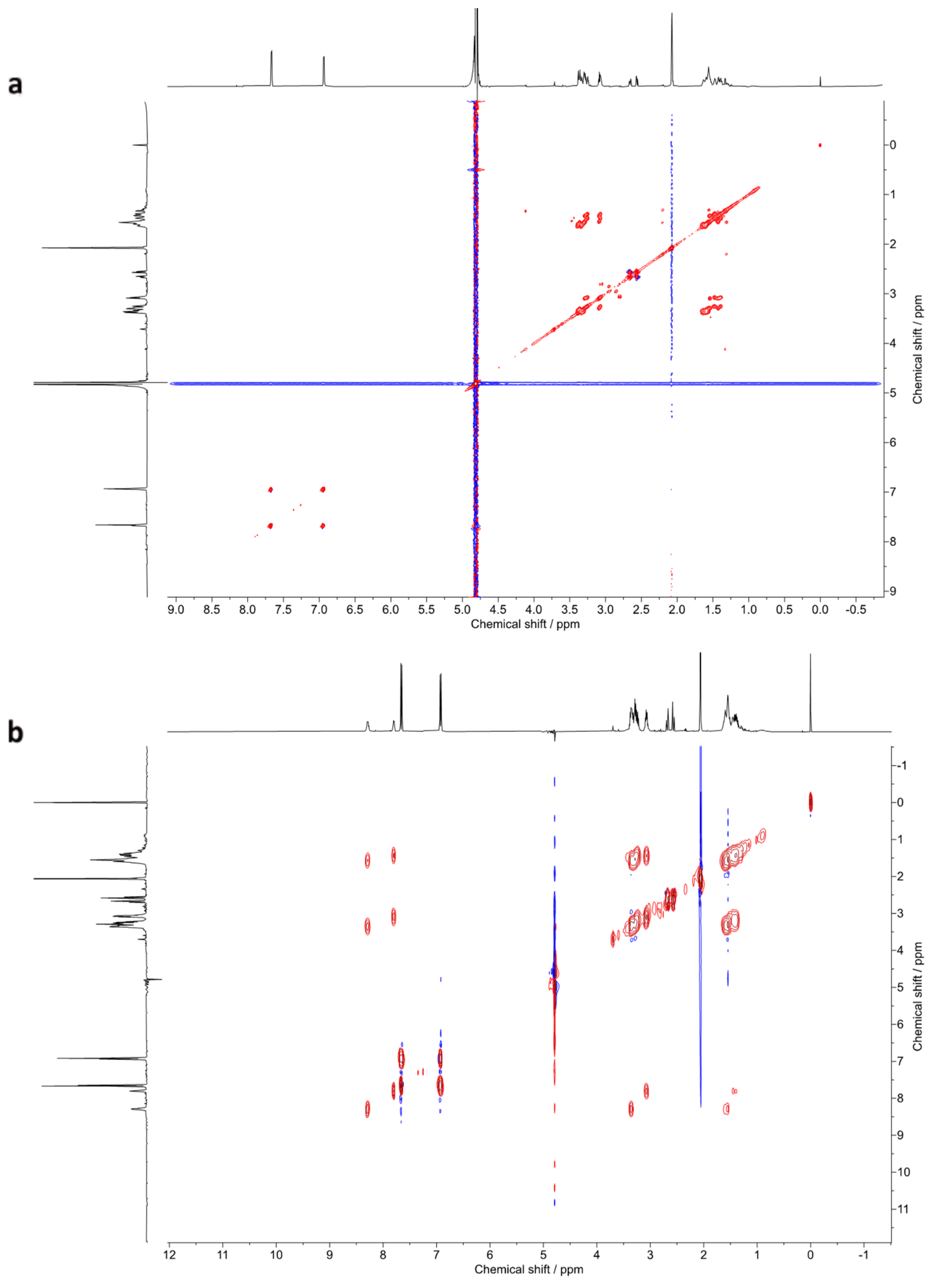

Figure S9. 2D proton NMR spectra of methylolanthanin. a, TOCSY spectrum (800 MHz, D_2O). **b**, TOCSY spectrum (500 MHz, 9:1 H_2O/D_2O) measured with water suppression.

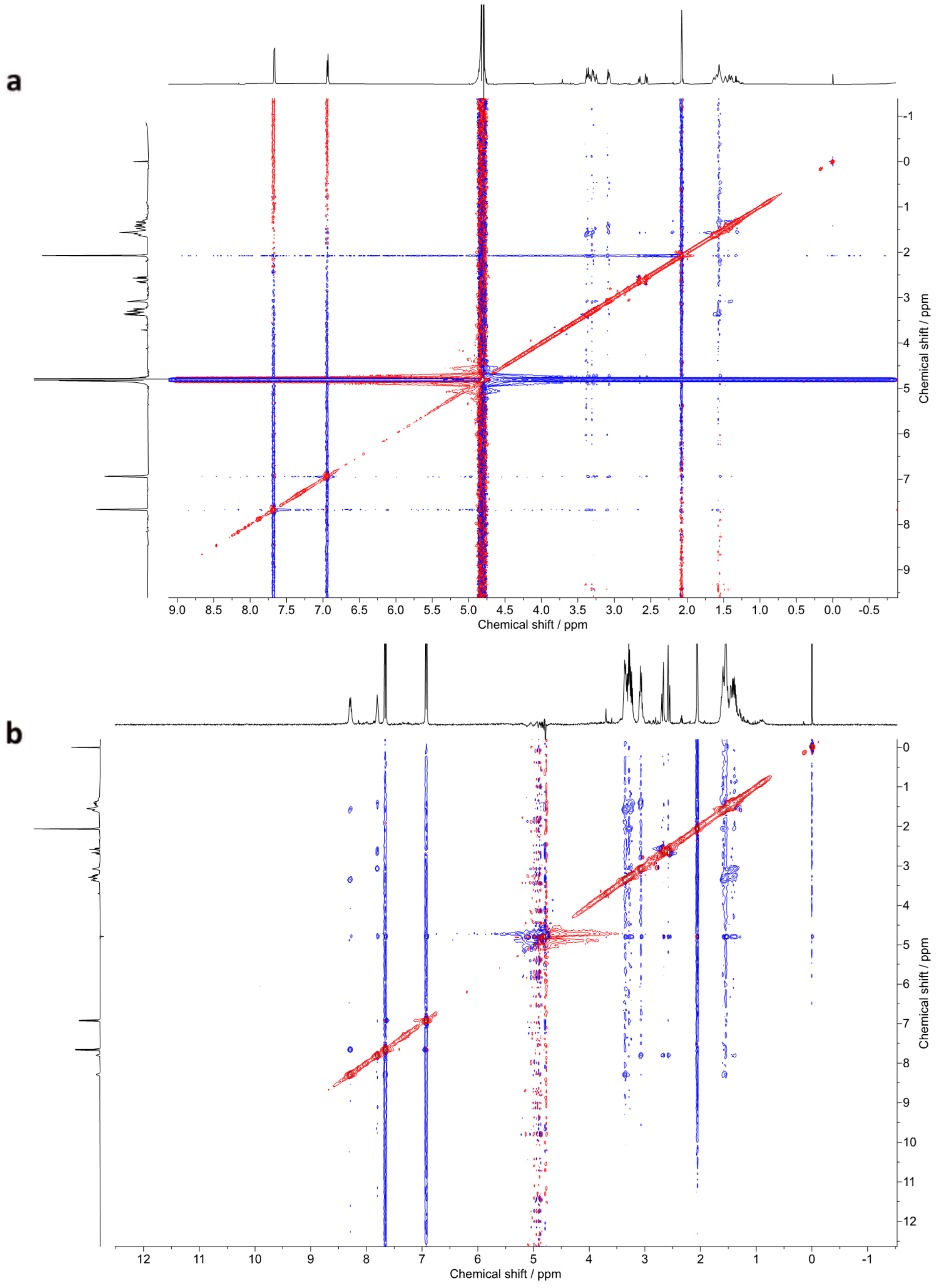

Figure S10. 2D proton NMR spectra of methylolanthanin. a, NOESY spectrum (800 MHz, D_2O). **b**, NOESY spectrum (500 MHz, 9:1 H_2O/D_2O) measured with water suppression.

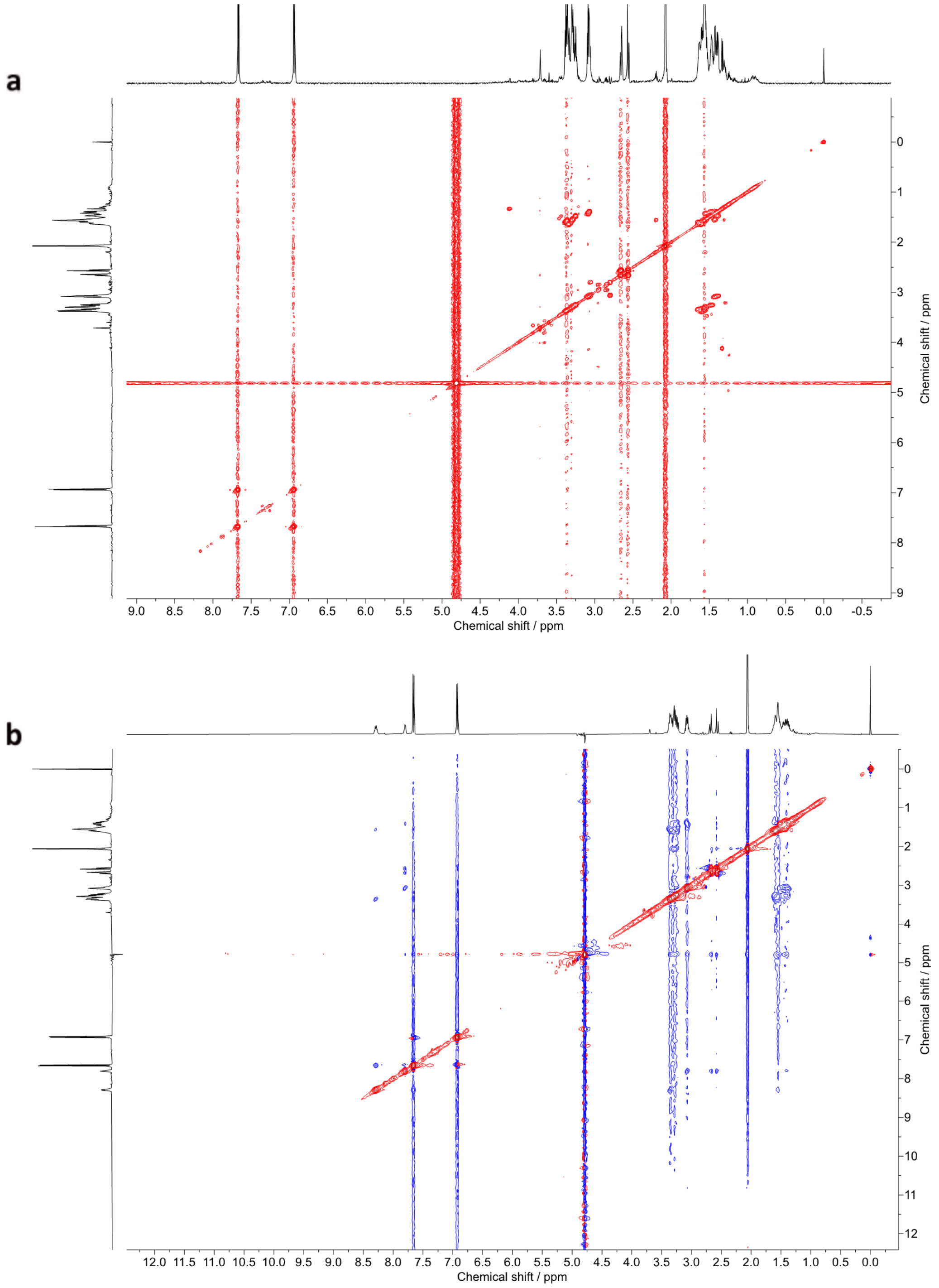

Figure S11. 2D proton NMR spectra of methylolanthanin. a, COSY spectrum (800 MHz, D_2O). **b**, ROESY (500 MHz, 9:1 H_2O/D_2O) spectrum measured with water suppression.

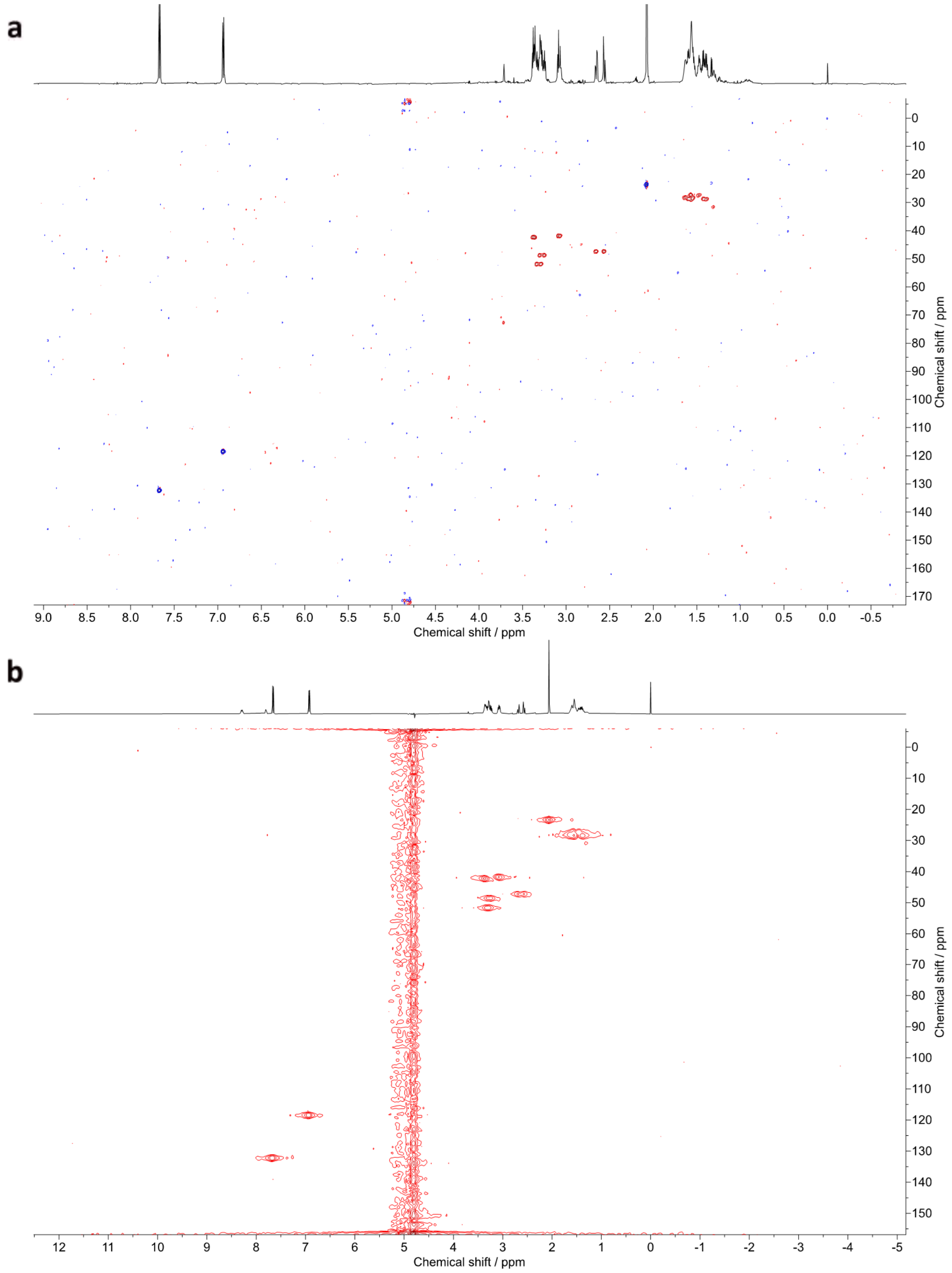

Figure S12. Heteronuclear ^{1}H-^{13}C 2D NMR spectra of methylolanthanin. **a**, Phase-sensitive HSQC (800 MHz, D_2O) spectrum **b**, HSQC (500 MHz, 9:1 H_2O/D_2O) spectrum measured with water suppression.

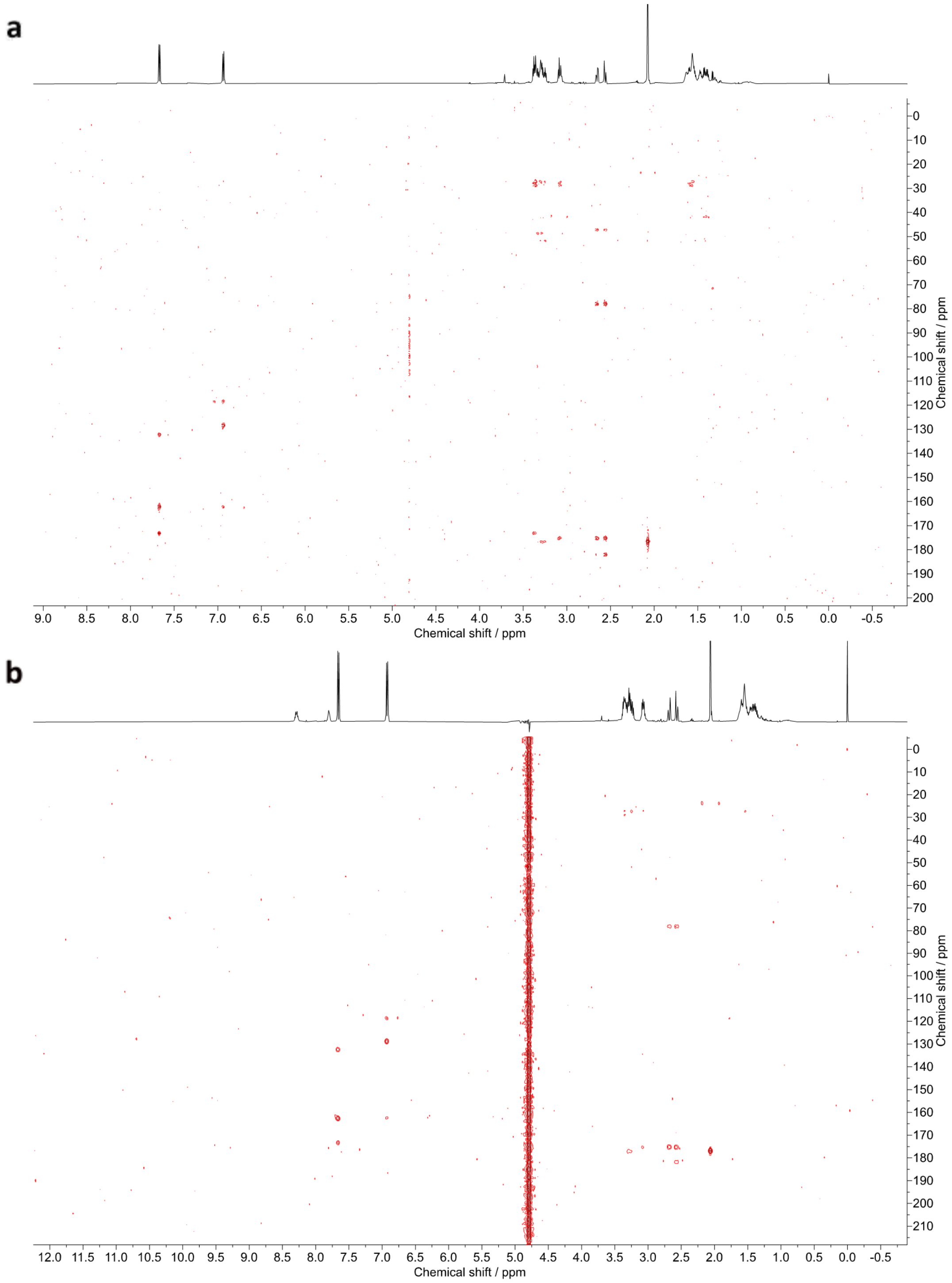

Figure S13. Heteronuclear ^{1}H-^{13}C 2D NMR spectra of methylolanthanin. a, HMBC (800 MHz, D_2O). **b**, HMBC (500 MHz, 9:1 H_2O/D_2O) spectrum measured with water suppression.

Supplementary Table 1. Prevalence of *mll* in *Methylobacteriaceae*

#Assembly	Strain	AsbABCDE present?	
GCF_001741865.1	Bosea vaviloviae Vaf18	False	
GCF_004802635.2	Methylocystis heyeri H2	False	
GCF_009363855.1	Microvirga thermotolerans HR1	False	
GCF_016804325.1	Methylobacterium aquaticum BG2	True	
GCF_001548015.1	Methylobacterium aquaticum MA-22A	False	
GCF_021228015.1	Methylobacterium currus TP-3	True	
GCF_003173715.1	Methylobacterium durans 17SD2-17	False	
GCF_029761955.1	Methylobacterium indicum JDJ13	False	
GCF_017347565.1	Methylobacterium indicum VL1	False	
GCF_000364445.2	Methylobacterium mesophilicum SR1.6/6	False	
GCF_029714205.1	Methylobacterium nodulans CB376	False	
GCF_000022085.1	Methylobacterium nodulans ORS 2060	False	
GCF_022533465.1	Methylobacterium organophilum WPA_B	False	
GCF_000757795.1	Methylobacterium oryzae CBMB20	False	
GCF_021398735.1	Methylobacterium oryzae H33R-06	False	

GCF_001936175.1	Methylobacterium phyllosphaerae CBMB27	False	
GCF_003173735.1	Methylobacterium radiodurans 17Sr1-43	False	
GCF_000019725.1	Methylobacterium radiotolerans JCM 2831	False	
GCF_021484845.1	Methylobacterium radiotolerans NYY1	False	
GCF_003173775.1	Methylobacterium sp. 17Sr1-1	False	
GCF_029691625.1	Methylobacterium sp. 391_Methyba4	False	
GCF_000019365.1	Methylobacterium sp. 4-46	False	
GCF_001542815.1	Methylobacterium sp. AMS5	True	
GCF_001854385.1	Methylobacterium sp. C1	False	
GCF_025813715.1	Methylobacterium sp. FF17	False	
GCF_028583545.1	Methylobacterium sp. NMS14P	False	
GCF_008000895.1	Methylobacterium sp. WL1	False	
GCF_003254375.1	Methylobacterium sp. XJLW	False	
GCF_023546765.1	Methylobacterium tardum DSM 19566	False	
GCF_003173755.1	Methylobacterium terrae 17Sr1-28	False	
GCF_022179725.1	Methylorubrum aminovorans NBRC 15686	True	

GCF_000022685.1	Methylorubrum extorquens AM1	True	
GCF_030062705.1	Methylorubrum extorquens ATCC 55366	True	
GCF_000021845.1	Methylorubrum extorquens CM4	True	
GCF_000083545.1	Methylorubrum extorquens DM4	True	
GCF_026122615.1	Methylorubrum extorquens NBC_00036	True	
GCF_026122595.1	Methylorubrum extorquens NBC_00404	True	
GCF_030255395.1	Methylorubrum extorquens PA1	True	
GCF_000018845.1	Methylorubrum extorquens PA1	True	
GCF_001971665.1	Methylorubrum extorquens PSBB040	True	
GCF_900234795.1	Methylorubrum extorquens TK 0001	**	Fragmented AsbC
GCF_022179745.1	Methylorubrum podarium DSM 15083	True	
GCF_000019945.1	Methylorubrum populi BJ001	True	
GCF_002355515.1	Methylorubrum populi P-1M	True	
GCF_006740745.1	Methylorubrum populi YC-XJ1	True	
GCF_014199985.1	Methylorubrum rhodesianum DSM 5687	True	
GCF_014199935.1	Methylorubrum rhodinum DSM 2163	False	

GCF_900114375.1	Methylorubrum salsuginis CGMCC 1.6474	False	
GCF_021117295.1	Methylorubrum sp. B1-46	True	
GCF_024347855.1	Methylorubrum sp. GM97	True	
GCF_022179765.1	Methylorubrum suomiense DSM 14458	False	
GCF_022179785.1	Methylorubrum thiocyanatum JCM 10893	True	
GCF_014845115.1	Methylorubrum zatmanii LMG 6087	False	

Supplementary Table 2. Primers used in study

Primer	Sequence (5' to 3')	Plasmid	Product
AZ09	ACGAGAAATCTTGCCGTTGGC	pMLL	META1p4132-META1p4133 + first 500 bp of META1p4134
AZ10	ATCAACCCGGATCTGCGCC	pMLL	Last 500 bp of META1p4133 + META1p4134-META1p4138
AZ11	GAGAACGAGATGACGTTGGA	pMLL	Joint fragment: 200 bp HZ848 backbone + 200 bp pCM66T
AZ12	ATTGCCATACCTTTGGTCGTTTTTTAGCCGCTAAAACGG	pMLL	Joint fragment: 200 bp HZ848 backbone + 200 bp pCM66T/HZ848 backbone
AZ13	CGACCAAAGGTATGGCAATTTAGAAAAACTCATCGAGCATCA	pMLL	Joint fragment: 200 bp HZ848 backbone + 200 bp pCM66T/pCM66T
AZ14	TCCTTTTAACAGCGATCGCG	pMLL	Joint fragment: 200 bp HZ848 backbone + 200 bp pCM66T
AZ15	CATTCGATAAGCCACGAAATATGACCATGATTACGCCAAG	pMLL	Joint fragment: 200 bp pCM66T + 200 bp EPI300 backbone
AZ16	TTTACCAAGGCATCATTGCATGTAAGCGGATGCCGG	pMLL	Joint fragment: 200 bp pCM66T + 200 bp EPI300 backbone/pCM66T
AZ17	TGCAATGATGCCTTGGTAAAATCTACGTCTGTCGAGAAGT	pMLL	Joint fragment: 200 bp pCM66T

			+ 200 bp EPI300 backbone/EPI300 backbone
AZ18	TTGATATCGGGGGTTAGTTCG	pMLL	Joint fragment: 200 bp pCM66T + 200 bp EPI300 backbone
AZ19	GTGTTCACCCTTGTTACACCGT	pMLL	Joint fragment: 200 bp pCM66T + 200 bp EPI300 backbone
AZ20	TGAATTGGACTTAACGATTCAGGGCACCAATAACTGCCT	pMLL	Joint fragment: 200 bp EPI300 backbone + first 200 bp META1p4132
AZ21	GAATCGTTAAGTCCAATTCAGATCATGCCACCCTCGTCTC	pMLL	Joint fragment: 200 bp EPI300 backbone + first 200 bp META1p4132/EPI300 backbone
AZ22	ATGAAGCCGGTTGCCGTATC	pMLL	META1p4132-META1p4133 + first 500 bp of META1p4134
AZ23	CTCGTTGGCGGTCTGGTCG	pMLL	Joint fragment: last 200 bp META1p4138 + 200 bp HZ848 backbone
AZ24	GTAGATTCGGCATTAGGCAACGGTTGCCACCTTTCGGGAC	pMLL	Last 500 bp of META1p4133 + META1p4134-META1p4138/joint fragment: last 200 bp META1p4138 + 200 bp HZ848
AZ25	TTGCCTAATGCCGAATCTACGAGGCCAAAAAGCTCGCTTTCAG	pMLL	Joint fragment: last 200 bp META1p4138+200 bp HZ848

			backbone/HZ848 backbone
AZ26	TGCCGGCACGTTAACCG	pMLL	Joint fragment: last 200 bp META1p4138 + 200 bp HZ848 backbone
AZ27	ATTTCGTGGCTTATCGAATGAACAAACCACCGCTGGTAGC	pMLL	AM1 backbone
MS25	ACTTGGTCTGACAGTTACCATCTCCCCGCATATCGACTAC	pMS2	*mll* upstream flank
MS26	TAGGAGCCGTGGATGTTAAG	pMS2	*mll* upstream flank
MS27	AACGTGACCATACGGGCCATTGAATCCCGGCAGCAAACCC	pMS2	*mll* downstream flank
MS28	GACACCGACAACCTGCACATTAGGAGCCGTGGATGTTAAGG	pMS2	*mll* downstream flank
MS29	CTTAACATCCACGGCTCCTAATGTGCAGGTTGTCGGTGTC	pMS2	pCM433kanT backbone
MS30	GTAGTCGATATGCGGGGAGATGGTAACTGTCAGACCAAGT	pMS2	pCM433kanT backbone
AZ195	TGATGCTCTAGACATGATCTTCGAAACCTCCTG	pAZ6	*mll* promoter
AZ196	GCTGCCGGGATTCAGGAGCCGCTGTTCATTCCGGTATTGGCT	pAZ6	*mll* promoter
